# Neuroinflammation, Sleep, and Circadian Rhythms

**DOI:** 10.3389/fcimb.2022.853096

**Published:** 2022-03-22

**Authors:** Mark R. Zielinski, Allison J. Gibbons

**Affiliations:** ^1^ Veterans Affairs (VA) Boston Healthcare System, West Roxbury, MA, United States; ^2^ Harvard Medical School, West Roxbury, MA, United States

**Keywords:** NLRP3 inflammasome, cytokines, electroencephalogram power, vagus nerve, neurovascular unit, inflammation

## Abstract

Molecules involved in innate immunity affect sleep and circadian oscillators and vice versa. Sleep-inducing inflammatory molecules are activated by increased waking activity and pathogens. Pathologies that alter inflammatory molecules, such as traumatic brain injury, cancer, cardiovascular disease, and stroke often are associated with disturbed sleep and electroencephalogram power spectra. Moreover, sleep disorders, such as insomnia and sleep disordered breathing, are associated with increased dysregulation of inflammatory processes. Inflammatory molecules in both the central nervous system and periphery can alter sleep. Inflammation can also modulate cerebral vascular hemodynamics which is associated with alterations in electroencephalogram power spectra. However, further research is needed to determine the interactions of sleep regulatory inflammatory molecules and circadian clocks. The purpose of this review is to: 1) describe the role of the inflammatory cytokines interleukin-1 beta and tumor necrosis factor-alpha and nucleotide-binding domain and leucine-rich repeat protein-3 inflammasomes in sleep regulation, 2) to discuss the relationship between the vagus nerve in translating inflammatory signals between the periphery and central nervous system to alter sleep, and 3) to present information about the relationship between cerebral vascular hemodynamics and the electroencephalogram during sleep.

## Introduction

Evidence of the involvement of immune-related molecules including those that are involved in inflammation in sleep regulation has increased over the past several decades (Opp and Krueger, 2015; Krueger et al., 2016; Zielinski et al., 2016; Besedovsky et al., 2019). Anecdotally, many people are aware of the relationship between infection and the immune system from disrupted sleep undergone from being infected by the common cold or influenza. When an individual remains awake late into the evening or is woken early in the morning without insufficient time to sleep, they are often aware of the resulting sleepiness. Additionally, individuals who travel across time zones become cognizant of the difficulty to sleep at their usual time or remain awake at times of the day when they are typically up and alert due to jet-lag. Research studies in humans and animals have uncovered specific immune and inflammatory molecules and mechanisms that regulate sleep and alter the circadian clock (Opp and Krueger, 2015; Krueger et al., 2016; Zielinski et al., 2016; Zielinski et al., 2017; Besedovsky et al., 2019). Research has also described how physiological mechanisms involving the vagus nerve and cerebral blood flow are involved with modulating sleep and the electroencephalogram (Zielinski et al., 2019; Williams and Lewis, 2020). Interestingly, a balance appears to occur between sleep and wake promoting molecules and vasoconstrictive and vasodilative molecules that affect sleep and wake states and the amplification of electroencephalogram power spectra, which are plausibly modulated by circadian oscillations (Zielinski et al., 2019). Herein, we describe the role of innate immune inflammatory molecules in sleep regulation and interactions with the circadian clock.

## Circadian Rhythms

Circadian rhythms occur at approximately 24 h in length corresponding to the earth rotation around the sun and function to align molecular, cellular, and behavioral activity (Patke et al., 2020). Light intensity from the sun or artificial sources are a major source of entrainment for circadian rhythms (Patke et al., 2020). Yet, other mechanisms of circadian rhythm entrainment exist including food, temperature, and exercise (Mistlberger RE, 1995; Refinetti, 2010; Pickel and Sung, 2020; Hughes et al., 2021). Light signals the retino-hypothalamic tract to activate neurons in the suprachiasmatic nuclei (SCN) located in the hypothalamus (Muindi et al., 2014). The SCN is a master pacemaker as it functions to entrain genes that govern circadian rhythms (Patke et al., 2020). In the absence of entrainment factors, circadian rhythms circadian synchronicity will drift over time under constant light or dark (Duffy and Czeisler, 2009). Circadian dysregulation is found for increased morbidity risk for inflammatory diseases including cardiovascular disease (Reutrakul and Knutson, 2015), cancer (Levi and Schibler, 2007), and metabolic disease (Arble et al., 2010). Circadian disruption is also prevalent with sleep disorders (Kim et al., 2013).

All cells have circadian rhythms and circadian clocks alter cell activity by transcriptional, posttranscriptional, translational, and posttranslational mechanisms to modify signaling pathways, metabolic activity, organelle functions, and the cell cycle (Chaix et al., 2016). For example, astrocytes within the brain are involved in promoting circadian rhythms from the SCN (Brancaccio et al., 2019). In organs and tissues, peripheral clocks are synchronized and coordinated by the SCN through the hypothalamic pituitary adrenal axis and the autonomic nervous system (Dibner et al., 2010). In mammals, a transcription-translational feedback loop is controlled by molecules that regulate circadian control processes (Patke et al., 2020). Circadian locomotor output cycles kaput (CLOCK) transcription factors and brain and muscle aryl hydrocarbon receptor nuclear translocator-like 1 (BMAL1) and bind E-box regulatory motifs to promote gene expression (Allada et al., 2001; Young and Kay, 2001; Takahashi, 2017). CLOCK-BMAL1 genes regulate the expression of the repressors period (PER) and cryptochromes (CRY) (Takahashi, 2017). PER and CRY proteins function to oligomerize and enter the nucleus to repress CLOCK-BMAL1 (Takahashi, 2017). BMAL1 expression timing and amplitude are mediated by competitive binding of Rev-Eebs, which are encoded by the nuclear receptor subfamily 1 group D member 1 (NR1D1) and member 2 (NR1D2) genes, resulting in repressing BMAL1 transcription or retinoic acid-related orphan receptor alpha (RORα) activation of BMAL1 transcription (Preitner et al., 2002; Sato et al., 2004). In addition, the albumin D-box binding protein (DBP) transcriptional activator and nuclear factor interleukin 3 regulated repressor act on PER and DBP to modulate their expression (Mitsui et al., 2001).

Several molecules that regulate or modulate sleep also alter the circadian clock and contrariwise. Inhibiting clock and period genes including CLOCK, BMAL1 PER1,PER2, PER3, CRY1, and CRY2 modifies homeostatic sleep (Albrecht, 2002). Mice lacking both CRY1 and CRY2 genes have impaired clock functions but interestingly have increased non-rapid-eye movement (NREM) sleep and electroencephalogram (EEG) delta power (0.5-4 Hz frequency range) occurring during NREM sleep [also referred to as slow-wave activity (SWA)] (Wisor et al., 2002). Additionally, PER1 and PER2 double knockout (KO) mice have increased SWA compared to control mice (Shiromani et al., 2004). Mice with a mutated CLOCK gene have reduced sleep when compared to wild-types (Naylor et al., 2000). In rats, cholinergic projections to the SCN from the pedunculopontine tegmentum (PPT) and laterodorsal tegmentum (LDT) suggest that acetylcholine activity in the brain can alter clock functions (Bina et al., 1993). Serotonergic projections to the SCN from the dorsal raphe also have the potential to alter clock functions (Moore et al., 1978). However, stronger evidence suggests that adenosine and glutamate, which are well known to regulate sleep, act on SCN clock functioning (Deboer, 2018). Findings also suggest that cellular activity in the SCN stimulates neurons in the ventrolateral preoptic nucleus to release the molecule noradrenaline that has potent arousal functions (Saint-Mleux et al., 2007)—which was observed from the long-lasting inhibition of norepinephrine from the selective alpha2-adrenoreceptor antagonist yohimbine. *In vitro* and *in vivo* studies using dopamine β-hydroxylase KO mice that do not produce norepinephrine or epinephrine show that in peripheral heart, liver, and white adipose tissue norepinephrine and epinephrine control clock gene, PER1, PER2, the basic leucine zipper transcriptional factor nuclear factor interleukin (IL)-3 also known as E4BP4, and DBP, although clock genes were preserved after chronic propanol and terazosin were administer suggesting these effects were not due to dopamine (Reilly et al., 2008). Nevertheless, evidence also suggests that circadian clocks are not altered by sleep deprivation, which is suggested by only small shifts or no changes in circadian phases in mice and hamsters (Mistlberger et al., 1983; Challet et al., 2001; van Diepen et al., 2014).

## Innate Immunity

The innate immune system is highly conserved between species (Riera Romo et al., 2016). The innate immune system is present in simple life forms and is largely used in more developed life forms such as vertebrates including rodents and humans (Riera Romo et al., 2016). The innate immune system functions include recruiting immune cells to infections sites, producing cell signaling molecules called cytokines, identification of foreign substances including bacteria, viruses, and protozoa, activation of complement cascades, clearing antibody complexes and dead cells, activating the adaptive immune system through antigen presentation of antigen presenting cells (APCs), altering the vascular system to protect the spread of pathogens or damaging substances, and regulates non-immunological functions such as cognition, and mood, and sleep (Zielinski and Krueger, 2011; Filiano et al., 2015; Riera Romo et al., 2016; Masih et al., 2019). Based on the visual observations of Celsius and Galen near the beginning of the common era, five cardinal signs of inflammation were identified including rubor (i.e., redness), tumor (i.e., swelling), calor (i.e., increased temperature), dolor (i.e., pain, and function laesa (i.e., loss of function) (Tracy, 2006). Notwithstanding, over the last two centuries, inflammation is now understood to be involved with many beneficial functions such as stimulating chemical substances to recruit cells or molecules to an injured cell, providing a physical barrier and response against pathogens, and also sleep (Zielinski and Krueger, 2011; Cui et al., 2014; Riera Romo et al., 2016). In this review, we discuss how inflammatory molecules and processes are critical to sleep regulation and the restorative functions of sleep aid in protection against pathogens and excessive increased activity. We also describe how chemical substances produced during inflammation affect sleep and SWA including nitric oxide, prostaglandins, energy-related molecules, and cytokines (Cui et al., 2014).

Cytokines are small protein or glycoprotein cell signaling molecules that are produced by nucleated cells (Oppenheim, 2001). Cytokines communicate through autocrine, paracrine, and endocrine mechanisms at very low concentrations, such as at picomolar levels (Zielinski and Krueger, 2011; García Morán et al., 2013). Cytokines function in immune responses, inflammation, and several physiological processes (Zielinski et al., 2019). A subset of cytokines called chemokines are major regulators of cell recruitment. Cytokines and chemokines are involved in regulating homeostatic sleep and sleep responses to sleep loss and infection (Zielinski et al., 2016; Zielinski et al., 2019). Cytokines in the periphery can also affect cytokines in the brain to affect sleep (Zielinski et al., 2019). Consequently, cytokines dysregulation in the brain is found with conditions that largely effect peripheral tissue and sleep such as cancer and cardiovascular disease (Williams et al., 2019) (Waldmann, 2018). Pro-inflammatory cytokines tend to promote sleep and SWA while anti-inflammatory cytokines, such as IL-4, IL-10, IL-13, and IL-1 receptor antagonist (RA), tend to attenuate sleep and SWA responses induced by sleep promoting stimuli including sleep deprivation, pathogens, orpathogenic components. Pro-inflammatory and anti-inflammatory molecules are expressed over different time courses which can potentially modulate the expression of each other (Zielinski and Krueger, 2011; Zielinski et al., 2016) IL-1 beta (IL-1β) and tumor necrosis factor-alpha (TNF-α) are the two most investigated pro-inflammatory cytokines that regulate sleep, and these molecules interact with the circadian system (Zielinski and Krueger, 2011; Zielinski et al., 2016). Nevertheless, many cytokines and chemokines are reported to modulate sleep or sleep responses to somnogenic stimuli (Zielinski and Krueger, 2011; Zielinski et al., 2016).

## IL-1β and Sleep

The IL-1 family of cytokines is currently defined by 11 members (IL-1α, IL-1β, IL-18, IL-33, IL-36α, IL-36β, IL-36 gamma (γ), IL-1RA, IL-36RA, IL-37, IL-38) which have both analogous and different effects (Xu et al., 2019). IL-1 family members function in immune responses, inflammation, and sleep (Zielinski and Krueger, 2011; Zielinski et al., 2016; Dinarello, 2018). Most IL-1 family members have pro-inflammatory actions, although IL-37 and IL-1RA have anti-inflammatory functions (Zielinski and Krueger, 2011; Dinarello, 2018). IL-1 family members are dysregulated in pathologies with disturbed sleep including cancer (Berger et al., 2005; Gelfo et al., 2020), brain damage (Viola-Saltzman and Watson, 2012), and cardiovascular disease (Boutin et al., 2001). Evidence in animal and human studies indicate that several IL-1 family members are shown to either regulate or modulate sleep including IL-1β, IL-1α, IL-18, and IL-37. The sleep altering actions of these IL-1 family members occur, in part, from the downstream activity of the receptors that they act upon (Zielinski and Krueger, 2011; Zielinski et al., 2016).

In rodents, IL-1β expression and protein levels in the cortex demonstrate diurnal patterns of activation with greater levels occurring during times of higher sleep propensity that happen at the beginning of the light period (Zielinski et al., 2017). On the one hand, these findings might suggest that the circadian clock controls the expression pattern of IL-1β in the brain. On the other hand, increased waking activity increases IL-1β levels suggesting that the diurnal variations might be largely attributed to increased local brain area use (Zielinski and Krueger, 2011; Zielinski et al., 2016). Sleep deprivation increases IL-1β expression and protein levels in the cortex and several other brain areas, peripheral tissue, and circulation in all species that have been investigated including rabbits, cats, monkeys, mice, rats, and humans (Zielinski and Krueger, 2011; Zielinski et al., 2016). Chronic sleep restriction also increases IL-1β in several brain areas including the cortex of rats (Zielinski et al., 2014). In rats, IL-1β expression in the hippocampus is increased after long terminal potentiation using tetanic stimulation (Balschun et al., 2003). Increased IL-1β immunoreactivity is also reported in corresponding barrel cortices after whisker stimulation further indicating the role of local use in inducing IL-1β activity in the brain of rats (Hallett et al., 2010). Infectious agents and their components also increase IL-1β in the brain of rodents and rabbits (Zielinski and Krueger, 2011; Zielinski et al., 2016). For example, when the gram-negative bacterial cell wall component lipopolysaccharide (LPS) is applied to the periphery of mice there is a resulting increase in IL-1β expression in the cortex (Zielinski et al., 2013). Also, influenza given to mice intranasally induces increased IL-1β expression in the hypothalamus (Zielinski et al., 2013). LPS, muramyl dipeptide, and influenza increase NREM sleep in rabbits, rats, mice, or humans (Zielinski and Krueger, 2011; Zielinski et al., 2016). These components of/or infectious agents also alter SWA (Zielinski and Krueger, 2011; Zielinski et al., 2016).

IL-1β applied centrally or to the periphery increases NREM sleep in all species (Zielinski and Krueger, 2011; Zielinski et al., 2016). IL-1β applied into the brainstem including the dorse raphe nuclei and the locus coeruleus increases NREM sleep amounts in rats and guinea pigs, respectively (De Sarro et al., 1997; Manfridi et al., 2003). Yet, often increased NREM sleep occurring after IL-1β administrate results in immediate reductions in rapid-eye movement (REM) sleep. Larger dosages of IL-1β can result in increased waking (Krueger et al., 2007). The reason for this effect is unknown but it could be from increased expression of anti-inflammatory cytokines including IL-4, IL-10, or IL-13 that can attenuate increased sleep after sleep promoting stimuli or sleep deprivation occurs, increased compensatory waking molecule induction, or discomfort (Zielinski and Krueger, 2011; Zielinski et al., 2016). IL-1β also largely increases SWA in most species (Zielinski and Krueger, 2011; Zielinski et al., 2016). Spontaneous sleep and sleep responses to sleep deprivation were attenuated after anti-IL1α and anti-IL-1β antibodies were given to rabbits (Obal et al., 1990). IL1β also can function to alter clock genes including CLOCK- BMAL1 activation of E-box regulatory elements, which may serve to alter spontaneous sleep and the normal homeostatic sleep responses to sleep promoting stimuli (Early et al., 2018; Timmons et al., 2021).

IL-1 receptor 1 (IL-1R1) and IL-1 receptor type 2 (IL-1R2) are two receptors for IL-1β and are found on a variety of cell types including, glia, neurons, epithelial cells, endothelial cells, macrophages, monocytes, neutrophils, T lymphocytes (Dinarello, 2018; Visan, 2019). IL-1 binding to the IL-1R1 leads to the activation of downstream signaling processes, although the IL-1R2 acts a decoy due to the lack of a signaling domain to prevent IL-1β signaling on the IL-1R1 (Dinarello, 2018). In addition, an IL-1RA exists that can bind to the IL-1R1 preventing IL-1β from acting on the IL-1R1 to induce downstream functions (Dinarello, 2018). IL-1R1 and the IL-1 receptor accessory protein (IL-1RAcP) form a heterodimer complex that allows for the signaling (Dinarello, 2018). The IL-1 receptor accessory protein b (IL-1RAcPb) also interacts with the IL-1R1 complex to inhibit the actions of IL-1RAcP (Gosselin et al., 2013). IL-1RAcPb is found predominantly in the brain on neurons (Gosselin et al., 2013). IL-1 receptor complex ectodomains attach to Toll/interleukin-1 receptor (TIR) domains within the cytoplasm. IL-1 receptor kinase (IRAK) adaptor molecule is associated with the TIR complex leading to myeloid differentiation primary response 88 (MYD88) signaling including c-Jun N-terminal kinase (JNK), p38 mitogen activated protein kinase (MAPK), and nuclear factor-kappa B (NF-κB) (Krumm et al., 2014; Dinarello, 2018).

NF-κB enters the nucleus and is involved in the transcription of cytokines and other molecules that regulate sleep, circadian rhythms, and immunity including IL-1β and TNF-α (Zielinski and Krueger, 2011; Liu et al., 2017; Hong et al., 2018). However, NF-κB also functions in the mitochondrial intermembrane space (Albensi, 2019). Activation of TNF-α and IL-1β receptors through their ligands results in NF-κB transcription (Liu et al., 2017). NF-κB activation involves intracellular processes that lead to the inhibitory-kappa B (I-κB) kinase complex to undergo phosphorylation (Liu et al., 2017). I-κB phosphorylation induces I-κB ubiquitination and degradation allowing NF-κB to translocate into the nucleus to induce the transcription of inflammatory molecules that can regulate sleep (Liu et al., 2017). NF-κB is regulated by several subunits that have either activation or repressor capabilities including p50, p52, p65, ribonucleic acid editing ligase A (RelA), ribonucleic acid editing ligase A (RelB), and c-terminal ribonculeic acid editing ligase (c-Re)l (Oeckinghaus and Ghosh, 2009). A diurnal variation in the expression of NF-κB has been reported to occur in the cortex of rodents with increased levels occurring during times of the day of increased sleep propensity (Chen et al., 1999). Increased waking activity from sleep loss increases NF-κB levels in the lateral hypothalamus, basal forebrain, and cortex in rodents and peripheral blood mononuclear cells in humans (Chen et al., 1999; Brandt et al., 2004; Ramesh et al., 2007; Irwin et al., 2008). Studies targeting NF-κB with KO mice or peptidergic inhibition indicate that NF-κB is involved in spontaneous sleep and sleep responses pathogens or their components. (Kubota et al., 2000; Ramkumar et al., 2011) For example, NF-ĸB p50 subunit KO mice have lower adenosine A1 and A2a receptors activation in the cortex and spontaneous sleep than control mice (Ramkumar et al., 2011). In mice, LPS acting through the Toll-like receptor 4 activation leads to NF-κB activation and the transcription of IL-1β and TNF-α (Liu et al., 2017). Mice lacking the NF-κB p50 subunits show reduced NREM sleep responses after LPS administration when compared to wild-type mice (Jhaveri et al., 2006). NF-κB p50 KO mice also have reduced NREM sleep responses to influenza infection compared to control mice further indicating the role of NF-κB in sleep regulation under homeostatic and pathogenic infection circumstances (Jhaveri et al., 2006).

An IL-RA reduced spontaneous NREM sleep and sleep responses muramyl dipeptide and IL-1β in rabbits (Opp and Krueger, 1991; Imeri et al., 1993). In rats, applying an IL-1R1 fragment intracerebroventricularly reduced NREM sleep (Takahashi et al., 1999). Mice lacking IL-1R1 have reduced NREM sleep and REM sleep amounts during the light period compared to wild-type mice (Fang et al., 1998; Huang, 2013). Mice lacking the IL-1R1 do respond to TNF-α with increased sleep (Fang et al., 1998). Furthermore, IL-1R1 and TNFR1 double KO mice have reduced NREM sleep and REM sleep rebounds after sleep deprivation (Baracchi and Opp, 2008). Sleep responses to LPS, influenza, and sleep deprivation also indicate a role of IL-1RAcP and IL-1RAcPb using transgenic mouse models (Taishi et al., 2012; Davis et al., 2015; Nguyen et al., 2019; Oles et al., 2020). Collectively, these findings indicate the somnogenic role of IL-1β on components of the IL-1 receptor in the brain.

**Figure 1:**
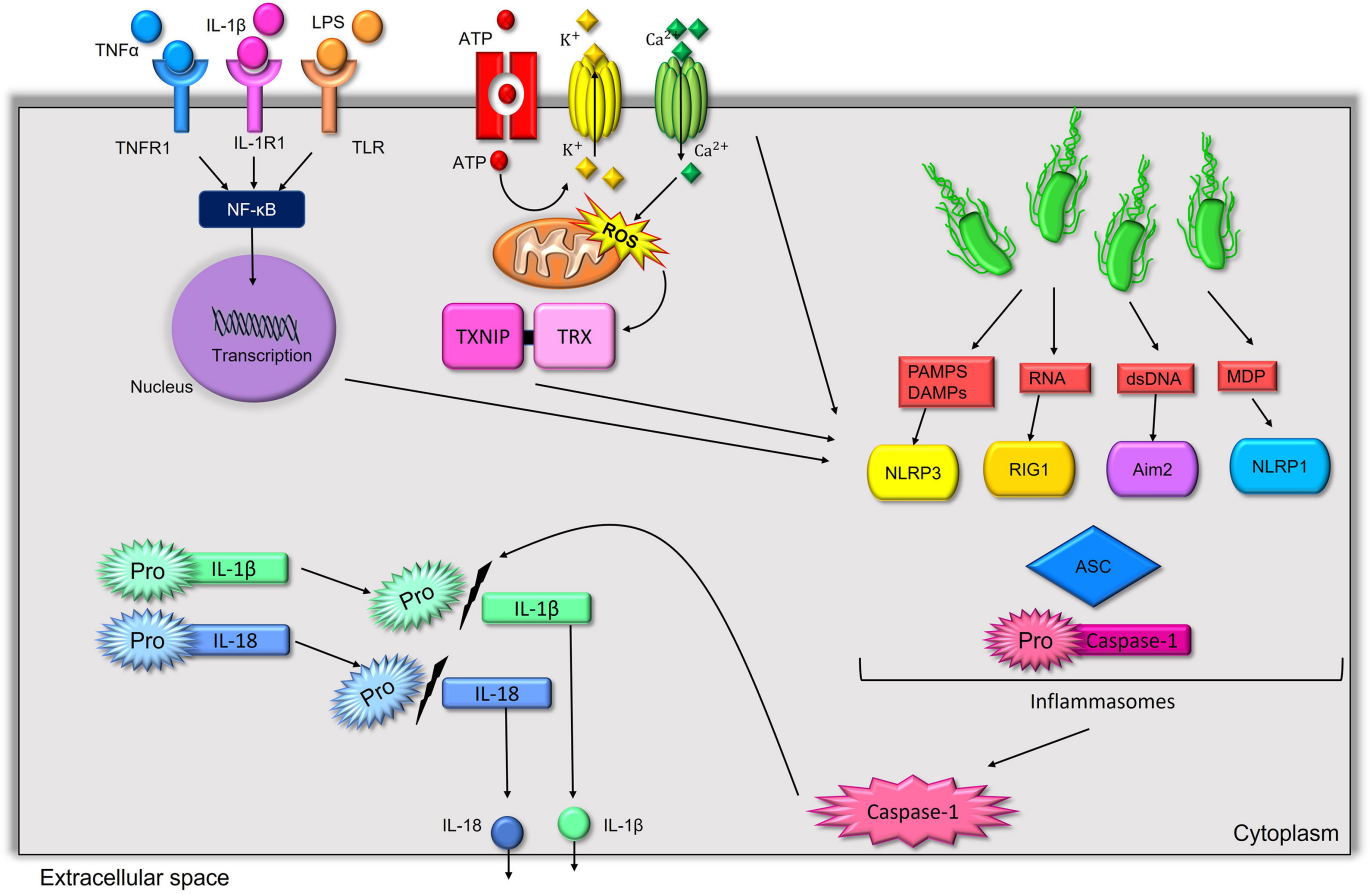
Major inflammasomes, their activators, and mechanisms that lead them to activate the somnogenic cytokines IL-1β and IL-18. NLRP3, RIG-1, AIM2, and NLRP1 are activated to combine with ASC and pro-caspase-1 to activate mature caspase-1, which will cleave the pro-forms of IL-1β and IL-18 into their mature active forms. NLRP3 is activated by multiple mechanisms including by extracellular ATP through the purine type 2 X7 receptor, oxidative stress involving TRX1 and TXNIP, and involves the priming of inflammasomes through NF-κB transcriptional processing of components of the inflammasome. This priming step can be activated by several somnogenic substances including LPS, IL-1β, and TNF-α through the TLR4, IL-1R1, and TNFR1, respectively.

Caspase-1 is the main enzyme that converts the pro-forms of IL-1β, IL-18, and IL-33 into their mature active forms (Sollberger et al., 2014). Additional molecules including elastase, chymases, granzyme A, cathepsin G, and proteinase-3, are also reported to cleave the pro-form of IL-1β into its mature form (Kaneko et al., 2019), although these molecules are currently not reported to have significant effects in glia and neurons. Inflammasomes are intracellular protein complexes that function to activate caspase-1 in response to specific danger associated molecular patterns (DAMPs) and pathogen associated molecular patterns (PAMPs) through their respective pattern recognition receptors (PRRs) (**Figure 1**). Inflammasomes are classified based upon nucleotide-binding oligomerization domain (NOD)-like receptors (NLRs), absent in melanoma 2 (AIM2), retinoic acid-inducible gene I (RIG-I), or pyrin which have specificity of activation based on their inducing factor (Voet et al., 2019; Li and Wu, 2021). Most inflammasomes have a apoptosis-associated speck-like protein containing a C-terminal caspase-recruitment domain (ASC) (also known as pycard1), which forms with their binding domains and pro-caspase-1 to form the inflammasome (Voet et al., 2019). The nucleotide-binding domain and leucine-rich repeat protein-3 (NLRP3) inflammasome is the most well-studied inflammasome and is involved in sleep regulation (Zielinski et al., 2017).

The NLRP3 inflammasome can be activated by a conical and a non-conical process (Pellegrini et al., 2017). The non-conical process is dependent on caspase-11 in mice and is dependent on caspase-4/5 in humans (Pellegrini et al., 2017). Non-conical NLRP3 inflammasome are activated by intracellular bacteria and bacteria cell wall components that function with caspase 1 to cleave a pore forming protein gasdermin-D to permeabilize the cell membrane and trigger a form of programmed cell death call pyroptosis (Pellegrini et al., 2017). The conical NLRP3 inflammasome is activated by a two-step process and does not always lead to apoptosis (Voet et al., 2019; Carty et al., 2019). The first step of NLRP3 inflammasome activation involves PRRs, such as the Toll-like 4 receptor activation by LPS or inflammatory molecule receptor activation, such as IL-1R1 or tumor necrosis factor receptor 2 (TNFR2) by IL-1β and TNF-α, respectively. The activation of these receptors leads to the activation of NF-κB to bring the transcription of components of the inflammasome and pro-forms of the cytokines that will be cleaved by caspase-1 (Zielinski et al., 2019). Evidence also suggests that the transcription factor activation protein-1 (AP-1) can be activated to transcribe components of the NLRP3 inflammasome (Zielinski et al., 2019). The second step of NLRP3 inflammasome activation uses energy-related molecules and oxidative stress components. Notably, extracellular adenosine tri-phosphate (ATP), which binds to purine type 2 receptors including the P2X7 receptor, can activate NLRP3 inflammasomes (Zielinski et al., 2019). Extracellular ATP activation involves a reduction of intracellular potassium levels and increased intracellular calcium levels to activate NLRP3 inflammasomes (Zielinski et al., 2019). The thioredoxin inhibitor interacting protein (TXNIP) is released by oxidative stress by the activation of the redox protein thioredoxin (TRX1) to activate NLRP3 inflammasomes and mitochondrial reactive oxygen species can activate the mechanistic target of rapamycin 1 (mTORC1) complex to increase the activation of NLRP3 inflammasomes indicating the role of oxidative stress on NLRP3 inflammasome activation (Zhou et al., 2010; Moon et al., 2015).

In mice, diurnal variations in caspase-1 activity, IL-1β protein levels, and NLRP3 and ASC gene expression are found in the somatosensory cortex with the greatest values occurring when there is high sleep propensity (Zielinski et al., 2017). These findings suggest that diurnal variations in IL-1β in the cortex occur, in part, from NLRP3 inflammasome activity. The caspase-1 inhibitor Ac-Tyr-Val-Ala-Asp chloromethyl ketone applied intracerebroventricularly reduced spontaneous NREM sleep in rats (Imeri et al., 2006). Cortical caspase-1 activity, IL-1β protein, levels, and caspase-1, NLRP3, ASC, and IL-1β gene expression are increased after sleep deprivation in wild-type but not NLRP3 KO mice (Zielinski et al., 2017). In mice, sleep deprivation also increases NLRP3 and IL-1β gene expression in the hippocampus. NLRP3 KO mice have attenuated spontaneous NREM sleep during the light period and lack the typical diurnal variation in SWA during spontaneous sleep (Zielinski et al., 2017). NLRP3 KO mice also have attenuated NREM sleep and sleep responses to sleep deprivation and LPS applied intracerebroventricularly compared to wild-type mice (Zielinski et al., 2017). Yet, NLRP3 KO mice show similar sleep responses after IL-1β is applied intracerebroventricularly, which is a molecule downstream of NLRP3 activation further indicating the role of NLRP3 inflammasome activity in the brain in sleep regulation (Zielinski et al., 2017). IL-18 infused centrally into rats, rabbits and mice promotes NREM sleep (Kubota et al., 2001; Zielinski et al., 2019). Alterations in the expression and immunoreactivity of IL-18 its receptor components IL18R1 and IL-18RAP were found in microglia within the somatosensory cortex, thalamus, and brainstem after sleep deprivation in wild-type mice but not NLRP3 KO mice (Gerashchenko et al., 2018; Johnston et al., 2018). Mice lacking IL-18 have attenuated NREM sleep and SWA responses to sleep deprivation and LPS compared with controls, although they have similar increased NREM sleep and SWA responses to centrally applied IL-18 protein (Zielinski et al., 2019). Together, these findings suggest that NLRP3 inflammasomes somnogenic effects can come from inducing both IL-1β and IL-18.

## TNF-α and Sleep

The TNF family consists of 19 members and 29 related receptors (Dostert et al., 2019). TNF-α is the most widely studied TNF family member and has well-established sleep regulatory functions (Rockstrom et al., 2018). TNF-α is produced by most nucleated cells including neurons and glia (Probert, 2015). TNF-α is involved in inflammation, immune functioning, cell survival, proliferation, and differentiation, cognition, mood and fatigue (Zielinski et al., 2019). Altered TNF-α expression is associated with pathologies that have dysregulated sleep and SWA including cancer, major depression, cardiovascular disease, and stroke (Zielinski et al., 2019). Elevated TNF-α levels are also associated with sleep apnea in humans (Cao et al., 2020).

The tumor necrosis factor converting enzyme (TACE), also known as a disintegrin and metalloprotease 17 (ADAM 17), functions to cleaves TNF-α into a soluble form that can bind to the TNF-α receptors—tumor necrosis factor receptor 1 (TNFR1) and TNFR2 (Sedger and McDermott, 2014). These receptors are found on most cells including immune cells, endothelial cells, glia, and neurons (Sedger and McDermott, 2014; Probert, 2015). TNFR1 has affinity for both the soluble and membrane forms of TNF-α but the TNFR2 has a higher affinity for its soluble form (Wajant and Siegmund, 2019). TNF-α activation is involved in cell death, which occurs, in part, by a death domain associated with TNFR1 (Wajant and Siegmund, 2019). TNFR2 does not contain the death domain (Wajant and Siegmund, 2019). Both TNF receptors can activated NF-κB, AP-1, and MAPK signaling (Wajant and Siegmund, 2019). TNF-α is also regulated translationally by a UA-rich sequence in the 3′ untranslated region in TNFα messenger RNA (Dean et al., 2001).

The exact mechanisms of how TNF-α is induced during homeostatic sleep remain unknown, although TNF-α is known to be involved in both sleep regulation and circadian biology. In rats, TNF-α protein levels in the cortex, hippocampus, and hypothalamus are higher during the beginning of the light period when sleep propensity is greatest (Floyd and Krueger, 1997). TNF-α can alter CLOCK-BMAL1 activation suggesting that circadian rhythms are altered can be modulated by TNF-α (Cavadini et al., 2007). Moreover, TNF-α shows daily rhythms in peripheral organs suggesting that peripheral clocks have the capability to alter TNF-α levels (Keller et al., 2009). Notwithstanding, in rodents, acute sleep deprivation or chronic sleep restriction increases TNF-α expression in the cortex, hippocampus, and brainstem suggesting the diurnal variation in TNF-α occurs, in part, from local activity use (Zielinski et al., 2014; Zielinski et al., 2016). Although the length of time that TNF-α is expressed differs from IL-1β and other cytokines, the effect of TNF-α increasing NREM sleep at the expense of REM sleep often occurs similar to that seen with IL-1β (Shoham et al., 1987; Kapas and Krueger, 1992). In rats, applying TNF-α to specific brain areas such as the locus coeruleus or the preoptic area of the anterior hypothalamus increases NREM sleep (De Sarro et al., 1997; Kubota et al., 2002). In mice, LPS applied to the peritoneum increases TNF-α expression in the cortex and nucleus tractus solitarius (NTS) (Zielinski et al., 2013). TNF-α expression is also increased in the hypothalamus after influenza is administer intranasally (Zielinski et al., 2013). Sleep increases after recombinant TNF-α is infused centrally in rabbits (Shoham et al., 1987). TNF-α applied intraperitoneally also increases NREM sleep in mice (Zielinski et al., 2013).

Experimental strategies targeting TNF-α and its receptors demonstrate their role in homeostatic sleep and sleep responses to pathogens. Antibodies targeting TNF-α or the TNF soluble receptor given to rodents attenuates increased sleep found after sleep deprivation (Takahashi et al., 1995). TNFR1 KO mice have lower spontaneous NREM and REM sleep and reduced sleep responses to TNF-α (Fang et al., 1997). However, TNFR1 KO mice respond normally to IL-1β with increased NREM sleep suggesting that IL-1β does not have a function in driving sleep by activating TNF-α. Mice lacking both TNF receptors have reduced SWA responses to influenza infection compared to control mice (Kapás et al., 2008). In rabbits given a TNF receptor fragment, their sleep responses to TNF-α and muramyl dipeptide were attenuated (Takahashi et al., 1996). Nevertheless, one study showed that TNF-α KO mice do not show difference in sleep and SWA responses to sleep deprivation from controls suggesting that effects of TNF-α are orchestrated by other sleep regulatory pathways such as NLRP3 inflammasomes (Szentirmai and Kapás, 2019).

## IL-1β and TNF-α Effects on Glutamate and Gamma-Aminobutyric Acid (GABA)

The exact mechanisms responsible for how inflammatory cytokines such as IL-1β and TNF-α affect neurons to induce sleep are unknown. However, much evidence indicates that glutamate/GABA signaling is imperative to sleep and wakefulness. TNF-α and IL-1β can induce glutamatergic activity (Furukawa and Mattson, 1998; De et al., 2003; De et al., 2003). TNF-α (Rindflesch et al., 2018) can modulate the glutamate receptor and synaptic scaling (Wang et al., 2012). The α-amino-3-hydroxy-5-methyl-4-isoxazolepropionic acid (AMPA) receptor is a subtype of the ionotropic glutamate receptor coupled to ion channels. AMPA receptor potentials are induced by TNF-α (Beattie et al., 2002), and calcium conductance is involved from AMPA receptor voltage-dependent mechanisms (Furukawa and Mattson, 1998; De et al., 2003; Stellwagen and Malenka, 2006). TNF-α can also increase the post-synaptic membrane (Beattie et al., 2002). Both TNF-α and IL-1β increase intracellular and extracellular glutamate levels (Ye et al., 2013; Zumkehr et al., 2018). TNF-α and IL-1β can also increase the glial glutamate transporter 1 which can facilitate glutamatergic transmission. (Ye et al., 2013; Zumkehr et al., 2018) IL-1β also modulates AMPA receptor expression and phosphorylation in neurons (Lai et al., 2006). Nevertheless, multiple mechanisms are likely involved of how cytokines alter molecules that can alter sleep and interact with circadian biology and indicative of the necessity of sleep. For example, TNF-α transient effect on inhibiting gene expression of the melatonin precursor Aa-nat, hiomt and synthesis of the melatonin precursor N-acetyl-serotonin in the pineal gland of rats (Fernandes et al., 2006).

## Adenosine and ATP and Sleep

ATP has been hypothesized to be involved in sleep regulation (Benington and Craig Heller, 1995). Some studies suggest that ATP is reduced in brain cells with increased wakefulness (Dworak et al., 2010), and other evidence suggests that sustained wakefulness increases extracellular levels of ATP and adenosine (Krueger et al., 2010). A major function of ATP is to store energy and transfer it within a cell but ATP is released from presynaptic neurons and can also act as a neurotransmitter (Khakh and North, 2012). Extracellular ATP is involved in the induction of inflammatory signaling (Burnstock, 2006). Nucleotide and nucleoside release stimulates ATP to enter the extracellular space (Idzko et al., 2014). Pannexins and connexins are involved in ATP signaling and ATP binds to purine type 2X and type 2Y receptors (Makarenkova et al., 2018). As previously mentioned, the P2X7 receptor is involved in NLRP3 inflammasome activation (Pelegrin, 2021). In rats, P2X7 receptor expression varies with times of the day where greater levels are seen when sleep propensity is greater (Krueger et al., 2010). Pharmacologically inhibiting the P2X7 receptor attenuates sleep responses to sleep deprivation in rats (Krueger et al., 2010). A pharmacological agonist of the P2X7 receptor applied centrally increases spontaneous sleep and SWA in rats (Krueger et al., 2010). P2X7 receptor KO mice have reduced NREM sleep and SWA responses to sleep deprivation. P2X7 receptor expression in the cortex is also reduced after sleep deprivation in mice (Krueger et al., 2010).

CD39 converts ATP to adenosine di-phosphate (ADP) and adenosine mono-phosphate (AMP) (Zielinski et al., 2012). Mice lacking the rate limiting enzyme, CD73, which converts AMP to adenosine have reduce NREM sleep and SWA responses to sleep deprivation suggesting that adenosine has sleep promoting effects after increased waking activity (Zielinski et al., 2012). A well-described molecule that is involved with regulating sleep/wakefulness is adenosine (Basheer et al., 2004; Bjorness and Greene, 2009). Adenosine acts through its receptors, especially the adenosine A1 and A2a receptors to affect sleep. In cats, extracellular adenosine levels are increased in the cortex and basal forebrain with increased waking activity (Porkka-Heiskanen et al., 1997; Porkka-Heiskanen et al., 2000). The adenosine A1 receptor works, in part, through neuron in the basal forebrain to promote wakefulness (Basheer et al., 2004; Bjorness and Greene, 2009). In rats, adenosine A2a receptors can activate GABAergic neurons in the ventrolateral preoptic nucleus that is located in the hypothalamus to promote sleep (Scammell et al., 2001; Kumar et al., 2013). In addition, adenosine A2a receptor KO mice have reduced sleep responses to sleep deprivation demonstrating the sleep promoting effects of the adenosine A2a receptor (Urade et al., 2003).

## Cyclooxygenase-Prostaglandin Pathway and Sleep

An additional major inflammatory pathway that is involved with modulating sleep and SWA is the cyclooxygenase (COX)-prostaglandin pathway (Huang et al., 2007; Zielinski and Krueger, 2011). COX is the rate-limiting enzyme that converts arachidonic acid to prostaglandin H2 (Ricciotti and FitzGerald, 2011). COX-2 is normally express at low levels but an inducible form functions in inflammation (Melikian et al., 2009). COX-2 is an inducible form of COX that is found in most cells including neurons and glia (Temel and Kahveci, 2009). COX-1 is a form of COX that is constrictively active and express on most cells (Fitzpatrick, 2004). COX-2 is induced by inflammatory and physiological stimuli and growth factors (Simon, 1999). In rodents, COX-2 expression is increased in astrocytes and microglia after intracerebroventricularly applied LPS (Font-Nieves et al., 2012). LPS induces COX-2 and prostaglandin E2 synthase-1, the enzyme that generates prostaglandin E from prostaglandin H2, in part, by MyD88-dependent NF-κB and MAPK pathways (Font-Nieves et al., 2012). Prostaglandin E2 is a vasoactive that as vasodilative properties. COX-2 can also be induced by IL-1β and TNF-α (Aïd and Bosetti, 2011). In rabbits, spontaneous sleep and sleep responses to TNF-α applied to the basal forebrain are reduced with COX-2 inhibition (Yoshida et al., 2003). Prostaglandin D2 synthase leads to the conversion of prostaglandin H2 from arachidonic acid and is increased in the brain after prolonged wakefulness (Huang et al., 2007). Prostaglandin D2 can activate adenosine A2a receptors and inhibit the histaminergic arousal system, and these mechanisms likely contribute to the sleep enhancing effect of prostaglandins (Huang et al., 2007). Evidence suggests that the somnogenic effects of prostaglandin D2 occur from the actions of the prostaglandin EP4 receptor (Yoshida et al., 2000). Additionally, prostaglandin E2 has been shown to suppress wakefulness (Onoe et al., 1992). The arousal effects of prostaglandin E2 appear to function, in part, from the activation of the prostaglandin EP1 and EP2 receptors in the posterior hypothalamus where the histaminergic tuberomammillary nucleus is located (Yoshida et al., 2000). Notwithstanding, non-steroidal anti-inflammatory agents that reduce COX and downstream prostaglandins only have modest effects on sleep in humans (Murphy et al., 1994).

Nitric oxide (NO) acts to induce inflammation and increase blood flow by causing local vasodilation (Chen et al., 2008). NOsynthase (NOS) serves to catalyze arginine and nicotinamide adenine dinucleotide phosphate (NADPH) and dioxygen to produce NO (Chen et al., 2008). There are three forms of NOS including neuronal NOS (nNOS), endothelial NOS (eNOS) and inducible NOS (iNOS) that are produced by neurons, endothelial cells, and microglia in the brain (Costa et al., 2016). Arginine is produced from citrulline in arginine and proline metabolism and consumes ATP in the process (Pols et al., 2021). Citrulline is produced by several mechanisms including from the byproduct of arginine and NOS and glutamine and glutamate (Swamy et al., 2010). Mouse KO models, pharmacological studies, and optogenetics indicate that NO, nNOS, iNOS, and eNOS can increase sleep and/or SWA (Zielinski et al., 2016). Microdialysis experiments indicated that iNOS and NO levels are increased in the frontal cortex and basal forebrain of sleep deprived rats (Kalinchuk et al., 2010; Kalinchuk et al., 2011). In rats, inhibiting NO with after L-nitro-arginine methyl ester (L-NAME) reduces sleep deprivation increases in NREM and REM sleep (Kapás et al., 1994). Mice lacking iNOS have less spontaneous NREM sleep (Chen et al., 2003). The activity of iNOS in cells is increased in the frontal cortex and basal forebrain of rats after sleep deprivation as assessed by immunoreactivity to iNOS and c-Fos antibodies (Kalinchuk et al., 2010; Kalinchuk et al., 2011). nNOS knockout mice have reduced homeostatic SWA sleep responses to sleep deprivation (Morairty et al., 2013).

In rodents, a novel subset of GABAergic interneurons that co-express neuropeptide Y, somatostatin, and the neurokinin-1 receptor located in the cortex, the caudate-putamen, olfactory bulb, corpus callosum and amygdala are activated during sleep occurring after prolonged wakefulness are correlated with increased SWA (Gerashchenko et al., 2008). When specifically targeting nNOS in somatostatin positive cells using a cross-sectional breeding strategy that largely inhibits the expression of nNOS neurons in the cortex, these mice primarily exhibited affects, albeit small, at the lower end of SWA frequency spectrum (i.e., < 1.5 Hz) (Zielinski et al., 2019). Using immunohistochemistry, chronic sleep restricted rats were found to continue to have activated cortical nNOS cells during sleep yet they did not have increased SWA suggesting that adaptations occur with chronic sleep loss that might limit their effect on SWA (Zielinski et al., 2013). Substance P is an inflammatory molecule that acts on the neurokinin-1 receptor and is found throughout most of the brain (Zielinski et al., 2015). Local administration of a NK-1R inhibitor and a substance P agonist to the cortex of mice attenuated or enhanced SWA, respectively, suggesting that cortical nNOS cells play a role in altering SWA (Zielinski et al., 2015)., However, the activation of neurokinin-1 receptors leads to the activation of pro-inflammatory cytokines that could also alter SWA (Zielinski et al., 2015).

## Vagus Nerve, Cytokines in the Brain, and Sleep

**Figure 2:**
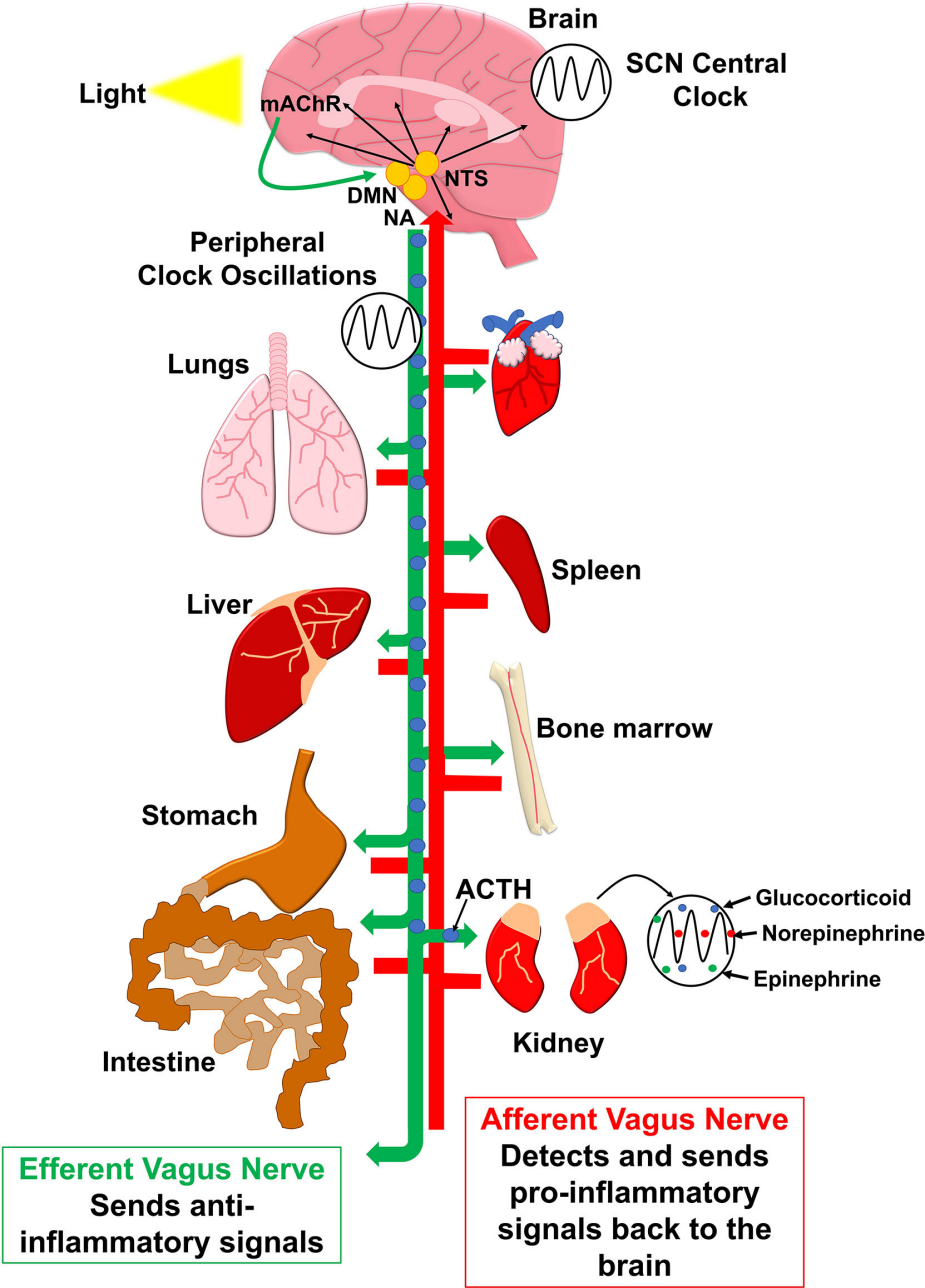
Pro-inflammatory and reductions in inflammatory responses occur in the brain and periphery, respectively, and are controlled by the vagus nerve. The vagal afferents can relay inflammatory stimuli from peripheral viscera to stimulate the NTS of the brain stem. The NTS projects to multiple sleep regulatory brain areas, which can induce pro-inflammatory somnogenic molecules and actions. The vagal efferents have anti-inflammatory actions that, in part, occur from acetylcholine receptor activation in the dorsal motor nucleus (DMN) and nucleus ambiguus (NA) to stimulate the vagal efferents to reduce inflammatory molecules in the periphery. The SCN plays a role in modulating the circadian actions of molecules in the brain and periphery. Additionally, peripheral clocks can affect these peripheral molecules that potentially can affect actions of vagal stimulation. Moreover, circadian clocks can alter glucocorticoids and epinephrine and norepinephrine, which can further modulate inflammatory responses to affect signaling between the periphery and brain.

Inflammatory molecules in the periphery can induce inflammatory molecules in the brain by traversing through leaky areas of the blood-brain-barrier such as the circumventricular organs including the subfornical organ, area postrema, vascular organ of the lamina terminalis, median eminence, pituitary gland, and pineal gland (Gross, 1992; Miyata, 2015), or stimulating the vagal afferent nerves (**Figure 2**) (Zielinski et al., 2019). The longest nerve in the autonomic nervous system is the tenth cranial nerve which is the vagus nerve. The vagus nerve has parasympathetic control of the viscera and mediates oxygen demand by altering respiratory control of the diaphragm, lungs, and heart (Bordoni et al., 2018). Vagal afferent stimulation can act to translate signals from the viscera from their projections to the dorsal vagal complex which involves the NTS, the dorsal motor nucleus (DMN), and the area postrema that is in the medulla area of the brainstem (Zielinski et al., 2019). The NTS projects to the amygdala, cortex, central nucleus of the amygdala, nucleus accumbens, paraventricular nucleus, and lateral hypothalamic areas of the hypothalamus, cerebellum, and other areas of the brainstem which all can affect sleep (García-Medina and Miranda, 2013). However, the vagal efferents can relate signals from the brainstem to organs in the periphery resulting in an attenuation in inflammation (Pavlov and Tracey, 2012).

In rodents that have their vagus nerve severed (i.e., vagotomized), NREM sleep and SWA are typically attenuated after IL-1β, TNF-α, or LPS is applied intraperitoneally (Hansen and Krueger, 1997; Kubota et al., 2001; Zielinski et al., 2013). The sleep modularly effects of the vagal afferents occur, in part, through the translation of inflammatory signals between the brain and periphery. Pro-inflammatory molecules including IL-1β and TNF-α or LPS, which induces IL-1β and TNF-α, administered to the peritoneum increase IL-1β and TNF-α expression in the cortex, hypothalamus, and NTS by stimulating the vagal afferents (Laye et al., 1995; Hansen et al., 1998; Zielinski et al., 2013). Mice and rats that have vagotomies have attenuated IL-1β and TNF-α expression in the brain after IL-1β, TNF-α, or LPS is applied to the peritoneum (Zielinski et al., 2013) (Laye et al., 1995) (Hansen et al., 1998). The effects of peripheral inflammation stimulating brain inflammatory molecules tend to occur through the vagal afferents at lower concentrations of inflammatory stimuli but at greater concentrations the effects of the vagotomy are reduced suggesting that inflammatory molecules are increased in the brain through leaky areas of the blood-brain-barrier in the circumventricular organs (Pavlov and Tracey, 2012; Zielinski et al., 2013; Kaur and Ling, 2017).

## Cerebral Vascular Hemodynamics and SWA

The relationship between SWA and sleep amount need is strong regarding sleep occurring after acute sleep deprivation (Davis et al., 2011). Independent mechanisms that can influence sleep/wakefulness states have consistently been described regarding sleep/SWA and circadian clocks, there is not always a distinction between SWA and sleep need (Davis et al., 2011). This effect is seen in rats that have the SCN lesioned have increased NREM sleep and SWA following sleep deprivation (Tobler et al., 1983; Trachsel et al., 1992; Wisor et al., 2002; Larkin et al., 2004). The lack of distinction between NREM sleep and SWA is also seen in mice with the genetic inhibition of circadian clocks or altering circadian clocks with light pulses results in normal homeostatic sleep responses (Borbély et al., 2016). The two-process model of sleep regulation incorporates SWA and sleep need as one of the two arms mediating sleep/wake states—the other being circadian factors (Borbély et al., 2016). However, much evidence indicates that sleep need and SWA are regulated by independent mechanisms (Davis et al., 2011). For example, benzodiazepines increase sleep but reduce SWA (Dijk, 2010). In addition, LPS or TNF-α applied to the peritoneum of mice induces marked increases in NREM sleep amounts but reductions in SWA (Zielinski et al., 2013). Moreover, chronic sleep restriction or sleep fragmentation in rats results in increased sleep amounts during spontaneous sleep following the restriction but SWA is not increased (Kim et al., 2007; Deurveilher et al., 2012; Zielinski et al., 2013).

The amplitude of electroencephalogram signals occurs, in part, from the sum of neuronal action potentials that are affected by synaptic scaling (Buzsáki et al., 2012). Neurotransmitters, ions, and numerous molecules including cytokines affect neuron signaling. Intracellular signaling mechanisms, neuronal projections, intracellular shuttles, receptor expression and density, and extracellular molecular concentrations, and clearance pressure of the molecules can all potentially serve to alter local neuronal action potentials and thus the amplitude of slow waves (Buzsáki et al., 2012). The extracellular space is a fluid filled space that is external to cell membranes and involves interstitial space between cells, blood vessels, perivascular spaces, and ventricular and subarachnoid spaces within the brain (Nicholson and Hrabětová, 2017). The extracellular space contains ions that aid in cellular signaling including maintaining resting and action potentials to allow the release of neurotransmitters from synapses by volume transmission (Nicholson and Hrabětová, 2017). Diffusion, tortuosity, and bulk flow, which is mainly confined to the perivascular face by glymphatic clearance, are two mechanisms that mediate molecule flow through the extracellular space are altered by cerebral blood flow (Nicholson and Hrabětová, 2017). Cerebral blood flow function to supply the brain with oxygen, nutrients, and signaling molecules to maintain homeostasis (Daneman and Prat, 2015). Intriguingly, growing evidence in humans and rodents indicates that cerebral blood flow is associated with change in SWA (Gerashchenko and Matsumura, 1996; Hofle et al., 1997; Tüshaus et al., 2017; Turner et al., 2020).

**Figure 3:**
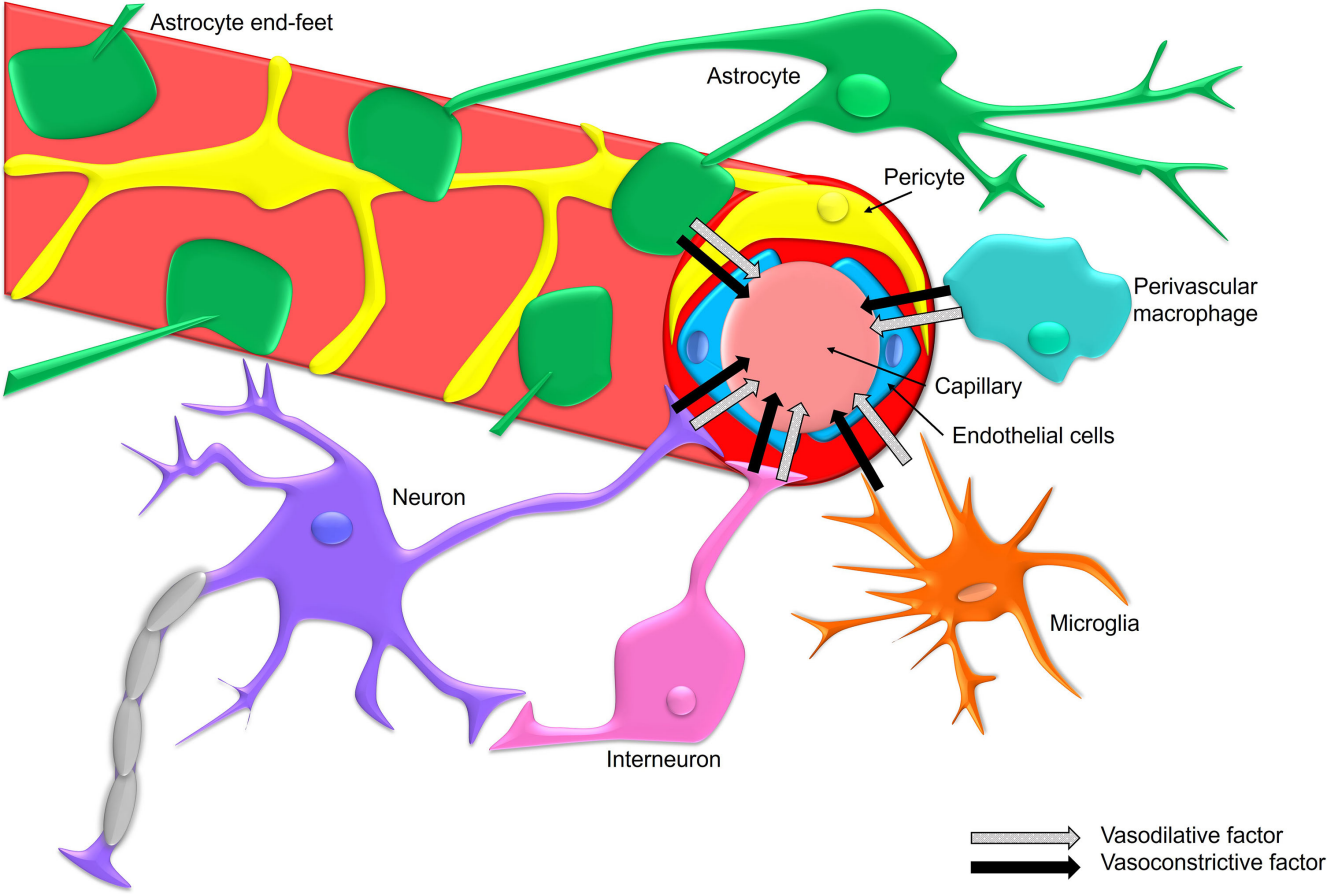
The neurovascular unit (NVU) tightly and rapidly regulates homeostasis in the brain by controlling the cerebral microvasculature. The neurovascular unit is comprised of endothelial cells, pericytes, astrocytes with end-feet that encompass the vasculature, neurons, interneurons, perivascular macrophages, and surrounding microglia. The NVU regulates blood flow in the brain in order to maintain the need from local use. The NVU also functions to sense local changes in the environment and responds to maintain homeostasis. The NVU acts in immunosurveillance to respond to potential pathogenic challenge or energy demand changes and responds with the release of inflammatory molecules. Sleep regulatory pro-inflammatory molecules can vasodilate to increase cerebral blood flow while other molecules, while many wake promoting molecules produced by neurons and glia have vasoconstrictive functions. Thus, the NVU likely has a major role in modulating cerebral blood flow changes occurring during sleep/wake states.

Cerebral blood vessels are relatively close to neurons and average about 15 um to the center of the closest neuronal soma (Tsai et al., 2009). The extracellular space is dynamic, and changes occur with increased brain activity, sleep and pathologies (Syková and Nicholson, 2008; Xie et al., 2013; Nicholson and Hrabětová, 2017). Several cell types surround cerebral blood vessels and release substances into the extracellular space and around blood vessels that can modulate cerebral blood flow. These cell types include endothelial cells, pericytes, astrocytes with end-feet that encompass the vasculature, neurons, interneurons, perivascular macrophages, and surrounding microglia and comprise the neurovascular unit (**Figure 3**) (Zielinski et al., 2019). Astrocytes are key modulators of cerebral blood flow (Macvicar and Newman, 2015). Blood flows from higher pressure areas to lower pressure areas and the velocity inversely correlates to the cross-sectional area of the vessel (Zielinski et al., 2019). Cerebral blood flow is the product of blood velocity and blood volume. Consequently, as vessels dilate then cerebral blood flow increases and as blood vessels constrict then cerebral blood flow is reduced (Zielinski et al., 2019). Interestingly, many but not all sleep promoting molecules including adenosine, IL-1β, TNF-α, and NO are vasodilative (Zielinski et al., 2019). Wake promoting molecules such as monoamines like norepinephrine tend to be vasoconstrictive (Zielinski et al., 2019). There is relative consistency between vasoregulatory actions of sleep promoting molecules and cells with those that increase SWA. However, this tenet does not hold true for all molecules and cells. This could be due, in part, to multiple different molecules that are released from certain cell populations and the bulk actions of these molecules on their receptors and downstream pathways, and the overall balance of the summation of the local activities of all sleep and wake promoting substances. Changes in cerebrovascular resistance modulate blood vessel diameters to maintain constant blood flow by cerebral autoregulation (Fantini et al., 2016). Vessel compliance normally functions to allow the vessels to dilate when demands need it (Zamir et al., 2018); however, circumstances exist that can impair vessel compliance such as occurs with prolonged or chronic inflammatory states. This is seen with animal models studying the activation of IL-1β inducing cerebral blood flow (Boutin et al., 2001; Farkas et al., 2006). Yet, IL-1β given chronically intracerebroventricularly results in a reduction in cerebral blood flow (Maher et al., 2003; Farkas et al., 2006).

## Future Directions

A need exists to understand the exact mechanisms of how innate immune and inflammatory mechanisms affecting sleep and circadian systems interact. The complexity of these relationships is affected by physics and physiology that are local, regional, and transverse peripheral and central nervous systems. We have entered an exciting time in the fields of neuroscience where we can activate and inhibit specific cells and molecules using techniques such as clustered regularly interspaced short palindromic repeats (CRISPR), optogenetics, chemogenetics, and fiber photometry and molecular and immunological techniques including next-generation sequencing and cytometry by time of flight (CyTOF) that can assess cell specific activity of far greater markers than previously possible. Recent advances in genetic-wide-associated studies (GWAS) are providing insight into genes and molecules that are dysregulated in sleep/wake and circadian disorders. For example, a recent GWAS publication in humans indicated that molecules upstream and downstream of NLRP3 inflammasome activation are associated with oxidative saturation levels in sleep-disordered breathing (Cade et al., 2019). Consequently, the rapid development of experimental models can now be made from these studies and allow for new discoveries on inflammatory mechanisms that affect sleep/wake and circadian disorders.

## Conclusion

**Figure 4:**
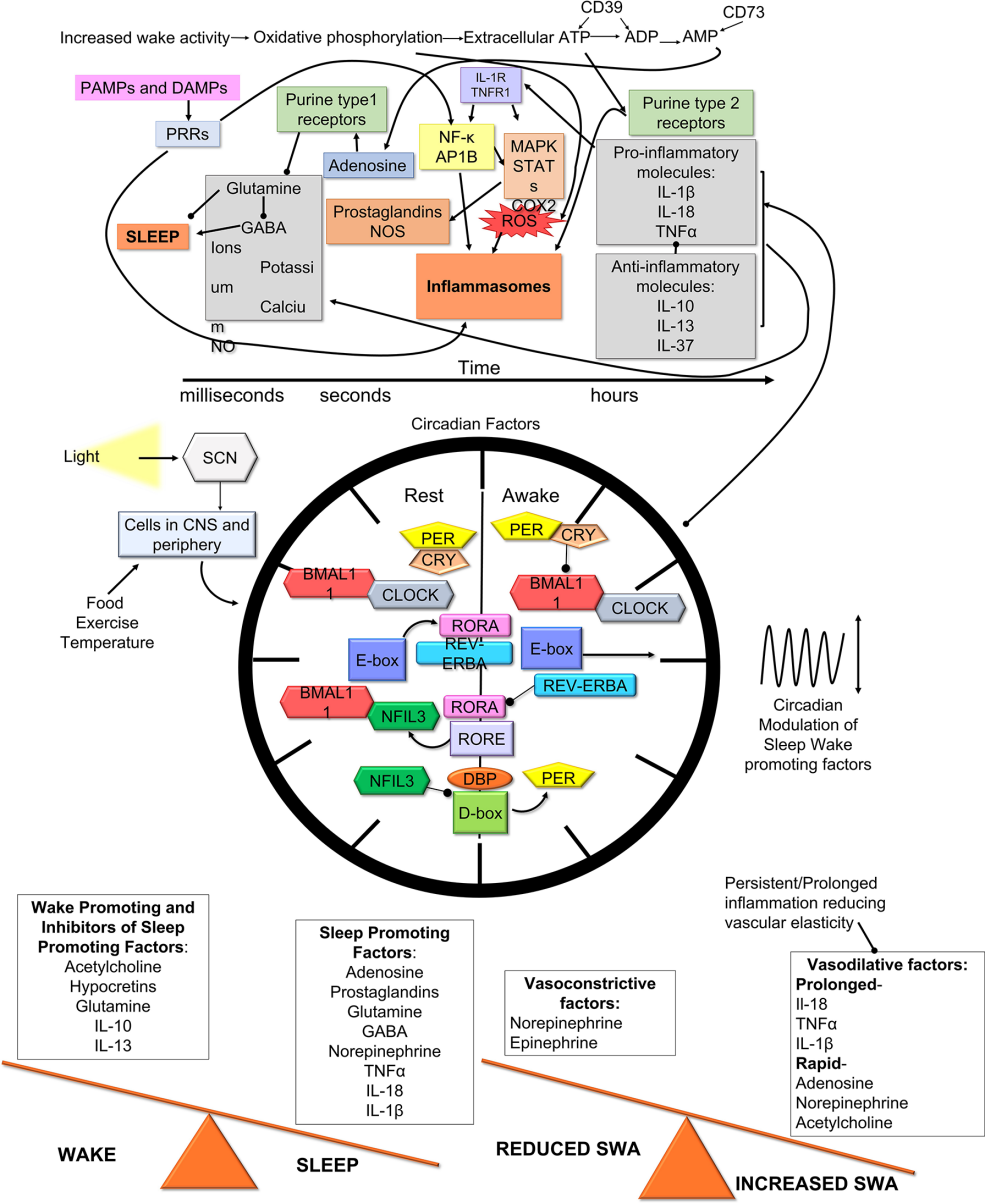
The Sleep, Slow-Wave, Vasohemodynamic Equipoise Hypothesis of Sleep Regulation. We hypothesize that sleep and SWA are regulated by specific inflammatory pathways and mechanisms that are modulated by or modulate circadian factors to enhance or diminish their effects. These inflammatory sleep regulatory molecules modulate neurotransmitters and physiological functions of the brains vasculature to affect sleep and/or SWA. Consequently, the summation of the effects on neurons and vasohemodynamics ultimately leads to localized changes in brain areas to induce sleep or alter SWA.

In summary, several inflammatory molecules and pathways can modulate sleep and SWA (Opp and Krueger, 2015; Krueger et al., 2016; Zielinski et al., 2016; Besedovsky et al., 2019) IL-1β and TNF-α function through their receptors to regulate sleep (Opp and Krueger, 2015; Zielinski et al., 2016; Krueger et al., 2016; Besedovsky et al., 2019). NLRP3 inflammasomes are critical sensing mechanisms that induces sleep and SWA in response to increased waking activity and pathogens (Zielinski et al., 2017). Increased evidence indicates that SWA and sleep need are independently regulated and alterations in vasohemodynamics likely is involved in altering SWA (Gerashchenko and Matsumura, 1996; Hofle et al., 1997; Davis et al., 2011; Tüshaus et al., 2017; Turner et al., 2020). Inflammatory molecules that regulate sleep can affect clock genes and vice versa, which likely contributes to an overall enhancement or suppression of sleep pressure that can induce or suppress sleep. Consequently, there appears to be a balance of circadian factors, inflammatory molecules, neurotransmitters, and physiological mechanisms governing vasohemodynamics that govern sleep regulation (**Figure 4**).

## Author Contributions

All authors listed have contributed to all aspects of this manuscript and made a substantial, direct, and intellectual contribution to the work, and approved it for publication.

## Funding

The preparation of the report was supported by the Department of Veterans Affairs grant IBX002823 (MZ).

## Conflict of Interest

The authors declare that the research was conducted in the absence of any commercial or financial relationships that could be construed as a potential conflict of interest.

## Publisher’s Note

All claims expressed in this article are solely those of the authors and do not necessarily represent those of their affiliated organizations, or those of the publisher, the editors and the reviewers. Any product that may be evaluated in this article, or claim that may be made by its manufacturer, is not guaranteed or endorsed by the publisher.

## References

[B1] Aïd S. Bosetti F. (2011). Targeting Cyclooxygenases-1 and -2 in Neuroinflammation: Therapeutic Implications. Biochimie 93 (1), 46–51. doi: 10.1016/J.BIOCHI.2010.09.009 20868723 PMC3008299

[B2] Albensi B. C. (2019). What Is Nuclear Factor Kappa B (NF-κb) Doing in and to the Mitochondrion? Front. Cell Dev. Biol. 7. doi: 10.3389/FCELL.2019.00154 PMC6692429 31448275

[B3] Albrecht U. (2002). Invited Review: Regulation of Mammalian Circadian Clock Genes. J. Appl. Physiol. 92 (3), 1348–1355. doi: 10.1152/JAPPLPHYSIOL.00759.2001 11842077

[B4] Allada R. Emery P. Takahashi J. S. Rosbash M. (2001). Stopping Time: The Genetics of Fly and Mouse Circadian Clocks. Annu. Rev. Neurosci. 24, 1091–1119. doi: 10.1146/ANNUREV.NEURO.24.1.1091 11520929

[B5] Arble D. M. Ramsey K. M. Bass J. Turek F. W. (2010). Circadian Disruption and Metabolic Disease: Findings From Animal Models. Best Pract. Res. Clin. Endocrinol. Metab. 24 (5), 785–800. doi: 10.1016/J.BEEM.2010.08.003 21112026 PMC3011935

[B6] Balschun D. Randolf A. Pitossi F. Schneider H. Del Rey A. Besedovsky H. O. (2003). Hippocampal Interleukin-1 Beta Gene Expression During Long-Term Potentiation Decays With Age. Ann. N. Y. Acad. Sci. 992, 1–8. doi: 10.1111/J.1749-6632.2003.TB03132.X 12794041

[B7] Baracchi F. Opp M. R. (2008). Sleep-Wake Behavior and Responses to Sleep Deprivation of Mice Lacking Both Interleukin-1β Receptor 1 and Tumor Necrosis Factor-α Receptor 1. Brain Behav. Immun. 22 (6), 982–993. doi: 10.1016/j.bbi.2008.02.001 18329246 PMC4164115

[B8] Basheer R. Strecker R. E. Thakkar M. M. McCarley R. W. (2004). Adenosine and Sleep-Wake Regulation. Prog. Neurobiol. 73 (6), 379–396. doi: 10.1016/J.PNEUROBIO.2004.06.004 15313333

[B9] Beattie E. C. Stellwagen D. Morishita W. Bresnahan J. C. Ha B. K. Von Zastrow M. (2002). Control of Synaptic Strength by Glial TNFalpha. Science 295 (5563), 2282–2285. doi: 10.1126/SCIENCE.1067859 11910117

[B10] Benington J. H. Craig Heller H. (1995). Restoration of Brain Energy Metabolism as the Function of Sleep. Prog. Neurobiol. 45 (4), 347–360. doi: 10.1016/0301-0082(94)00057-O 7624482

[B11] Berger A. M. Parker K. P. Young-McCaughan S. Mallory G. A. Barsevick A. M. Beck S. L.. (2005). Sleep Wake Disturbances in People With Cancer and Their Caregivers: State of the Science. Oncol. Nurs. Forum. 32 (6), E98-E126. doi: 10.1188/05.ONF.E98-E126 16270104

[B12] Besedovsky L. Lange T. Haack M. (2019). The Sleep-Immune Crosstalk in Health and Disease. Physiol. Rev. 99 (3), 1325–1380. doi: 10.1152/PHYSREV.00010.2018 30920354 PMC6689741

[B13] Bina K. G. Rusak B. Semba K. (1993). Localization of Cholinergic Neurons in the Forebrain and Brainstem That Project to the Suprachiasmatic Nucleus of the Hypothalamus in Rat. J. Comp. Neurol. 335 (2), 295–307. doi: 10.1002/CNE.903350212 8227520

[B14] Bjorness T. Greene R. (2009). Adenosine and Sleep. Curr. Neuropharmacol. 7 (3), 238–245. doi: 10.2174/157015909789152182 20190965 PMC2769007

[B15] Borbély A. A. Daan S. Wirz-Justice A. Deboer T. (2016). The Two-Process Model of Sleep Regulation: A Reappraisal. J. Sleep. Res. 25 (2), 131–143. doi: 10.1111/JSR.12371 26762182

[B16] Bordoni B. Purgol S. Bizzarri A. Modica M. Morabito B. (2018). The Influence of Breathing on the Central Nervous System. Cureus 10 (6), e2724. doi: 10.7759/cureus.2724 30083485 PMC6070065

[B17] Boutin H. LeFeuvre R. A. Horai R. Asano M. Iwakura Y. Rothwell N. J. (2001). Role of IL-1alpha and IL-1beta in Ischemic Brain Damage. J. Neurosci. 21 (15), 5528–5534. doi: 10.1523/JNEUROSCI.21-15-05528.2001 11466424 PMC6762680

[B18] Brancaccio M. Edwards M. D. Patton A. P. Smyllie N. J. Chesham J. E. Maywood E. S.. (2019). Cell-Autonomous Clock of Astrocytes Drives Circadian Behavior in Mammals. Science 363 (6423), 187–192. doi: 10.1126/SCIENCE.AAT4104 30630934 PMC6440650

[B19] Brandt J. A. Churchill L. Rehman A. Ellis G. Mémet S. Israël A.. (2004). Sleep Deprivation Increases the Activation of Nuclear Factor Kappa B in Lateral Hypothalamic Cells. Brain Res. 1004 (1-2), 91–97. doi: 10.1016/J.BRAINRES.2003.11.079 15033423

[B20] Burnstock G. (2006). Historical Review: ATP as a Neurotransmitter. Trends Pharmacol. Sci. 27 (3), 166–176. doi: 10.1016/J.TIPS.2006.01.005 16487603

[B21] Buzsáki G. Anastassiou C. A. Koch C. (2012). The Origin of Extracellular Fields and Currents–EEG, ECoG, LFP and Spikes. Nat. Rev. Neurosci. 13 (6), 407–420. doi: 10.1038/NRN3241 22595786 PMC4907333

[B22] Cade B. E. Chen H. Stilp A. M. Louie T. Ancoli-Israel S. Arens R.. (2019). Associations of Variants in the Hexokinase 1 and Interleukin 18 Receptor Regions With Oxyhemoglobin Saturation During Sleep. Montgomery CG, Ed. PloS Genet. 15 (4), e1007739. doi: 10.1371/journal.pgen.1007739 30990817 PMC6467367

[B23] Cao Y. Song Y. Ning P. Zhang L. Wu S. Quan J.. (2020). Association Between Tumor Necrosis Factor Alpha and Obstructive Sleep Apnea in Adults: A Meta-Analysis Update. BMC Pulm. Med. 20 (1), 1–17. doi: 10.1186/S12890-020-01253-0 32787816 PMC7425010

[B24] Carty M. Kearney J. Shanahan K. A. Hams E. Sugisawa R. Connolly D.. (2019). Cell Survival and Cytokine Release After Inflammasome Activation Is Regulated by the Toll-IL-1r Protein SARM. Immunity 50 (6), 1412–1424.e6. doi: 10.1016/J.IMMUNI.2019.04.005 31076360

[B25] Cavadini G. Petrzilka S. Kohler P. Jud C. Tobler I. Birchler T.. (2007). TNF-α Suppresses the Expression of Clock Genes by Interfering With E-Box-Mediated Transcription. Proc. Natl. Acad. Sci. U. S. A. 104 (31), 12843–12848. doi: 10.1073/pnas.0701466104 17646651 PMC1937554

[B26] Chaix A. Zarrinpar A. Panda S. (2016). The Circadian Coordination of Cell Biology. J. Cell Biol. 215 (1), 15–25. doi: 10.1083/JCB.201603076 27738003 PMC5057284

[B27] Challet E. Turek F. W. Laute M. A. Van Reeth O. (2001). Sleep Deprivation Decreases Phase-Shift Responses of Circadian Rhythms to Light in the Mouse: Role of Serotonergic and Metabolic Signals. Brain Res. 909 (1-2), 81–91. doi: 10.1016/S0006-8993(01)02625-7 11478924

[B28] Chen Z. Gardi J. Kushikata T. Fang J. Krueger J. M. (1999). Nuclear factor-kappaB-Like Activity Increases in Murine Cerebral Cortex After Sleep Deprivation. Am. J. Physiol. 276 (6), R1812–R1818. doi: 10.1152/AJPREGU.1999.276.6.R1812 10362764

[B29] Chen L. Majde J. A. Krueger J. M. (2003). Spontaneous Sleep in Mice With Targeted Disruptions of Neuronal or Inducible Nitric Oxide Synthase Genes. Brain Res. 973 (2), 214–222. doi: 10.1016/S0006-8993(03)02484-3 12738065

[B30] Chen K. Pittman R. N. Popel A. S. (2008). Nitric Oxide in the Vasculature: Where Does it Come From and Where Does it Go? A Quantitative Perspective. Antioxid. Redox Signal. 10 (7), 1185–1198. doi: 10.1089/ARS.2007.1959 18331202 PMC2932548

[B31] Costa E. D. Rezende B. A. Cortes S. F. Lemos V. S. (2016). Neuronal Nitric Oxide Synthase in Vascular Physiology and Diseases. Front. Physiol. 7. doi: 10.3389/FPHYS.2016.00206 PMC4889596 27313545

[B32] Cui J. Chen Y. Wang H. Y. Wang R. F. (2014). Mechanisms and Pathways of Innate Immune Activation and Regulation in Health and Cancer. Hum. Vaccin. Immunother. 10 (11), 3270–3285. doi: 10.4161/21645515.2014.979640 25625930 PMC4514086

[B33] Daneman R. Prat A. (2015). The Blood–Brain Barrier. Cold Spring Harb. Perspect. Biol. 7 (1), a020412. doi: 10.1101/cshperspect.a020412 25561720 PMC4292164

[B34] Davis C. J. Clinton J. M. Jewett K. A. Zielinski M. R. Krueger J. M. (2011). Delta Wave Power: An Independent Sleep Phenotype or Epiphenomenon? J. Clin. Sleep. Med. 7 (5), S16–S18. doi: 10.5664/JCSM.1346 22003323 PMC3190419

[B35] Davis C. J. Dunbrasky D. Oonk M. Taishi P. Opp M. R. Krueger J. M. (2015). The Neuron-Specific Interleukin-1 Receptor Accessory Protein is Required for Homeostatic Sleep and Sleep Responses to Influenza Viral Challenge in Mice. Brain Behav. Immun. 47, 35–43. doi: 10.1016/J.BBI.2014.10.013 25449578 PMC4418942

[B36] Dean J. L. E. Wait R. Mahtani K. R. Sully G. Clark A. R. Saklatvala J. (2001). The 3’ Untranslated Region of Tumor Necrosis Factor Alpha mRNA is a Target of the mRNA-Stabilizing Factor HuR. Mol. Cell Biol. 21 (3), 721–730. doi: 10.1128/MCB.21.3.721-730.2001 11154260 PMC86664

[B37] Deboer T. (2018). Sleep Homeostasis and the Circadian Clock: Do the Circadian Pacemaker and the Sleep Homeostat Influence Each Other’s Functioning? Neurobiol. Sleep. Circadian. Rhythm. 5, 68–77. doi: 10.1016/J.NBSCR.2018.02.003 PMC6584681 31236513

[B38] De A. Krueger J. M. Simasko S. M. (2003). Tumor Necrosis Factor Alpha Increases Cytosolic Calcium Responses to AMPA and KCl in Primary Cultures of Rat Hippocampal Neurons. Brain Res. 981 (1-2), 133–142. doi: 10.1016/S0006-8993(03)02997-4 12885434

[B39] De Sarro G. Gareri P. Sinopoli V. A. David E. Rotiroti D. (1997). Comparative, Behavioural and Electrocortical Effects of Tumor Necrosis Factor-α and Interleukin-1 Microinjected Into the Locus Coeruleus of Rat. Life Sci. 60 (8), 555–564. doi: 10.1016/S0024-3205(96)00692-3 9042390

[B40] Deurveilher S. Rusak B. Semba K. (2012). Time-of-Day Modulation of Homeostatic and Allostatic Sleep Responses to Chronic Sleep Restriction in Rats. Am. J. Physiol. - Regul. Integr. Comp. Physiol. 302 (12), R1411–R1425. doi: 10.1152/ajpregu.00678.2011 22492816

[B41] Dibner C. Schibler U. Albrecht U. (2010). The Mammalian Circadian Timing System: Organization and Coordination of Central and Peripheral Clocks. Annu. Rev. Physiol. 72, 517–549. doi: 10.1146/ANNUREV-PHYSIOL-021909-135821 20148687

[B42] Dijk D. J. (2010). Slow-Wave Sleep Deficiency and Enhancement: Implications for Insomnia and its Management. World J. Biol. Psychiatry 11 (Sup 1), 22–28. doi: 10.3109/15622971003637645 20509829

[B43] Dinarello C. A. (2018). Overview of the IL-1 Family in Innate Inflammation and Acquired Immunity. Immunol. Rev. 281 (1), 8–27. doi: 10.1111/imr.12621 29247995 PMC5756628

[B44] Dostert C. Grusdat M. Letellier E. Brenner D.. (2018). The TNF Family of Ligands and Receptors: Communication Modules in the Immune System and Beyond. Physiol. Rev. 99 (1), 115–160. doi: 10.1152/PHYSREV.00045.2017 30354964

[B45] Duffy J. F. Czeisler C. A. (2009). Effect of Light on Human Circadian Physiology. Sleep. Med. Clin. 4 (2), 165–177. doi: 10.1016/J.JSMC.2009.01.004 20161220 PMC2717723

[B46] Dworak M. McCarley R. W. Kim T. Kalinchuk A. V. Basheer R. (2010). Sleep and Brain Energy Levels: ATP Changes During Sleep. J. Neurosci. 30 (26), 9007–9016. doi: 10.1523/JNEUROSCI.1423-10.2010 20592221 PMC2917728

[B47] Early J. O. Menon D. Wyse C. A. Cervantes-Silva M. P. Zaslona Z. Caroll R. G.. (2018). Circadian Clock Protein BMAL1 Regulates IL-1β in Macrophages *via* NRF2. Proc. Natl. Acad. Sci. U. S. A. 115 (36), E8460–E8468. doi: 10.1073/PNAS.1800431115 30127006 PMC6130388

[B48] Fang J. Wang Y. Krueger J. M. (1997). Mice Lacking the TNF 55 kDa Receptor Fail to Sleep More After Tnfα Treatment. J. Neurosci. 17 (15), 5949–5955. doi: 10.1523/jneurosci.17-15-05949.1997 9221791 PMC6573218

[B49] Fang J. Wang Y. Krueger J. M. (1998). Effects of Interleukin-1β on Sleep are Mediated by the Type I Receptor. Am. J. Physiol. - Regul. Integr. Comp. Physiol. 274 (3 43-3), R655. doi: 10.1152/ajpregu.1998.274.3.r655 9530230

[B50] Fantini S. Sassaroli A. Tgavalekos K. T. Kornbluth J. (2016). Cerebral Blood Flow and Autoregulation: Current Measurement Techniques and Prospects for Noninvasive Optical Methods. Neurophotonics 3 (3), 031411. doi: 10.1117/1.nph.3.3.031411 27403447 PMC4914489

[B51] Farkas E. Süle Z. Tóth-Szuki V. Mátyás A. Antal P. Farkas I. G.. (2006). Tumor Necrosis Factor-Alpha Increases Cerebral Blood Flow and Ultrastructural Capillary Damage Through the Release of Nitric Oxide in the Rat Brain. Microvasc. Res. 72 (3), 113–119. doi: 10.1016/j.mvr.2006.05.007 16854437

[B52] Fernandes P. A. C. M. Cecon E. Markus R. P. Ferreira Z. S. (2006). Effect of TNF-Alpha on the Melatonin Synthetic Pathway in the Rat Pineal Gland: Basis for a “Feedback” of the Immune Response on Circadian Timing. J. Pineal. Res. 41 (4), 344–350. doi: 10.1111/J.1600-079X.2006.00373.X 17014691

[B53] Filiano A. J. Gadani S. P. Kipnis J. (2015). Interactions of Innate and Adaptive Immunity in Brain Development and Function. Brain Res. 1617, 18–27. doi: 10.1016/J.BRAINRES.2014.07.050 25110235 PMC4320678

[B54] Fitzpatrick F. A. (2004). Cyclooxygenase Enzymes: Regulation and Function. Curr. Pharm. Des. 10 (6), 577–588. doi: 10.2174/1381612043453144 14965321

[B55] Floyd R. A. Krueger J. M. (1997). Diurnal Variation of Tnfα in the Rat Brain. Neuroreport 8 (4), 915–918. doi: 10.1097/00001756-199703030-00020 9141064

[B56] Font-Nieves M. Sans-Fons M. G. Gorina R. Bonfill-Teixidor E. Salas-Peŕdomo A. Maŕquez-Kisinousky L.. (2012). Induction of COX-2 Enzyme and Down-Regulation of COX-1 Expression by Lipopolysaccharide (LPS) Control Prostaglandin E2 Production in Astrocytes. J. Biol. Chem. 287 (9), 6454–6468. doi: 10.1074/JBC.M111.327874 22219191 PMC3307308

[B57] Furukawa K. Mattson M. P. (1998). The Transcription Factor NF-kappaB Mediates Increases in Calcium Currents and Decreases in NMDA- and AMPA/kainate-Induced Currents Induced by Tumor Necrosis Factor-Alpha in Hippocampal Neurons. J. Neurochem. 70 (5), 1876–1886. doi: 10.1046/J.1471-4159.1998.70051876.X 9572271

[B58] García-Medina N. E. Miranda M. I. (2013). Nucleus of the Solitary Tract Chemical Stimulation Induces Extracellular Norepinephrine Release in the Lateral and Basolateral Amygdala. Brain Stimul. 6 (2), 198–201. doi: 10.1016/j.brs.2012.03.020 22543094

[B59] García Morán G. A. Parra-Medina R. Cardona A. G. Quintero-Ronderos P. Rodríguez É. G. (2013). Cytokines, Chemokines, and Growth Factors. in Autoimmunity: From Bench to Bedside [Internet]. Eds. Anaya J. M. Shoenfeld Y. Rojas-Villarga A.. Bugota (Colombia): El Rosario University Press. Available at: https://www.ncbi.nlm.nih.gov/books/NBK459450/ 29087650

[B60] Gelfo V. Romaniello D. Mazzeschi M. Sgarzi M. Grilli G. Morselli A.. (2020). Roles of IL-1 in Cancer: From Tumor Progression to Resistance to Targeted Therapies. Int. J. Mol. Sci. 21 (17), 1–14. doi: 10.3390/IJMS21176009 PMC7503335 32825489

[B61] Gerashchenko D. Matsumura H. (1996). Continuous Recordings of Brain Regional Circulation During Sleep/Wake State Transitions in Rats. Am. J. Physiol. - Regul. Integr. Comp. Physiol. 270 (4 39-4), R855. doi: 10.1152/ajpregu.1996.270.4.r855 8967416

[B62] Gerashchenko D. Niznikiewicz M. M. Johnston A. M. Basheer R. Strecker R. E. Zielinski M. R. (2018). 0032 Absence Of Nlrp3 Inflammasomes Reduces Cognitive Performance Impairments Induced By Sleep Loss. Sleep 41 (suppl_1), A13–A13. doi: 10.1093/SLEEP/ZSY061.031

[B63] Gerashchenko D. Wisor J. P. Burns D. Reh R. K. Shiromani P. J. Sakurai T.. (2008). Identification of a Population of Sleep-Active Cerebral Cortex Neurons. Proc. Natl. Acad. Sci. U. S. A. 105 (29), 10227–10232. doi: 10.1073/pnas.0803125105 18645184 PMC2481371

[B64] Gosselin D. Bellavance M. A. Rivest S. (2013). IL-1racpb Signaling Regulates Adaptive Mechanisms in Neurons That Promote Their Long-Term Survival Following Excitotoxic Insults. Front. Cell Neurosci. 7. doi: 10.3389/FNCEL.2013.00009 PMC3573345 23423359

[B65] Gross P. M. (1992). Circumventricular Organ Capillaries. Prog. Brain Res. 91, 219–233. doi: 10.1016/S0079-6123(08)62338-9 1410407

[B66] Hallett H. Churchill L. Taishi P. De A. Krueger J. M. (2010). Whisker Stimulation Increases Expression of Nerve Growth Factor- and Interleukin-1β-Immunoreactivity in the Rat Somatosensory Cortex. Brain Res. 1333, 48–56. doi: 10.1016/j.brainres.2010.03.048 20338152 PMC2879054

[B67] Hansen M. K. Krueger J. M. (1997). Subdiaphragmatic Vagotomy Blocks the Sleep-and Fever-Promoting Effects of Interleukin-1β. Am. J. Physiol. - Regul. Integr. Comp. Physiol. 273 (4 42-4), R1246–R1253. doi: 10.1152/ajpregu.1997.273.4.r1246 9362287

[B68] Hansen M. K. Taishi P. Chen Z. Krueger J. M. (1998). Vagotomy Blocks the Induction of Interleukin-1β (IL-1β) mRNA in the Brain of Rats in Response to Systemic IL-1β. J. Neurosci. 18 (6), 2247–2253. doi: 10.1523/jneurosci.18-06-02247.1998 9482809 PMC6792909

[B69] Hofle N. Paus T. Reutens D. Fiset P. Gotman J. Evans A. C.. (1997). Regional Cerebral Blood Flow Changes as a Function of Delta and Spindle Activity During Slow Wave Sleep in Humans. J. Neurosci. 17 (12), 4800–4808. doi: 10.1523/jneurosci.17-12-04800.1997 9169538 PMC6573353

[B70] Hong H. K. Maury E. Ramsey K. M. Perelis M. Marcheva B. Omura C.. (2018). Requirement for NF-κb in Maintenance of Molecular and Behavioral Circadian Rhythms in Mice. Genes Dev. 32 (21-22), 1367–1379. doi: 10.1101/GAD.319228.118/-/DC1 30366905 PMC6217733

[B71] Huang T. (2013). Sleep Alterations in the Interleukin-1 Type 1 Receptor Knockout Mice. Sleep. Med. 14, e155. doi: 10.1016/J.SLEEP.2013.11.356

[B72] Huang Z. L. Urade Y. Hayaishi O. (2007). Prostaglandins and Adenosine in the Regulation of Sleep and Wakefulness. Curr. Opin. Pharmacol. 7 (1), 33–38. doi: 10.1016/j.coph.2006.09.004 17129762

[B73] Hughes A. T. L. Samuels R. E. Baño-Otálora B. Belle M. D. C. Wegner S. Guilding C.. (2021). Timed Daily Exercise Remodels Circadian Rhythms in Mice. Commun. Biol. 4 (1), 761. doi: 10.1038/S42003-021-02239-2 34145388 PMC8213798

[B74] Idzko M. Ferrari D. Riegel A. K. Eltzschig H. K. (2014). Extracellular Nucleotide and Nucleoside Signaling in Vascular and Blood Disease. Blood 124 (7), 1029–1037. doi: 10.1182/BLOOD-2013-09-402560 25001468 PMC4133480

[B75] Imeri L. Bianchi S. Opp M. R. (2006). Inhibition of Caspase-1 in Rat Brain Reduces Spontaneous Nonrapid Eye Movement Sleep and Nonrapid Eye Movement Sleep Enhancement Induced by Lipopolysaccharide. Am. J. Physiol. - Regul. Integr. Comp. Physiol. 291 (1), R197–R204. doi: 10.1152/ajpregu.00828.2005 16455762

[B76] Imeri L. Opp M. R. Krueger J. M. (1993). An IL-1 Receptor and an IL-1 Receptor Antagonist Attenuate Muramyl Dipeptide- and IL-1-Induced Sleep and Fever. Am. J. Physiol. 265 (4 Pt 2), R197–204. doi: 10.1152/AJPREGU.1993.265.4.R907 8238464

[B77] Irwin M. R. Wang M. Ribeiro D. Cho H. J. Olmstead R. Breen E. C.. (2008). Sleep Loss Activates Cellular Inflammatory Signaling. Biol. Psychiatry 64 (6), 538–540. doi: 10.1016/J.BIOPSYCH.2008.05.004 18561896 PMC2547406

[B78] Jhaveri K. A. Ramkumar V. Trammell R. A. Toth L. A. (2006). Spontaneous, Homeostatic, and Inflammation-Induced Sleep in NF-kappaB P50 Knockout Mice. Am. J. Physiol. Regul. Integr. Comp. Physiol. 291 (5), R1516–R1526. doi: 10.1152/AJPREGU.00262.2006 16793936

[B79] Johnston A. M. Niznikiewicz M. M. Gerashchenko D. Strecker R. E. Basheer R. Zielinski M. R. (2018). 0031 Nlrp3 Inflammasome Mediates Il-18 And Il-18 Receptor Responses To Sleep Loss. Sleep 41 (suppl_1), A13–A13. doi: 10.1093/SLEEP/ZSY061.030

[B80] Kalinchuk A. V. McCarley R. W. Porkka-Heiskanen T. Basheer R. (2010). Sleep Deprivation Triggers Inducible Nitric Oxide-Dependent Nitric Oxide Production in Wake-Active Basal Forebrain Neurons. J. Neurosci. 30 (40), 13254–13264. doi: 10.1523/JNEUROSCI.0014-10.2010 20926651 PMC3496746

[B81] Kalinchuk A. V. McCarley R. W. Porkka-Heiskanen T. Basheer R.. (2011). The Time Course of Adenosine, Nitric Oxide (NO) and Inducible NO Synthase Changes in the Brain With Sleep Loss and Their Role in the non-Rapid Eye Movement Sleep Homeostatic Cascade. J. Neurochem. 116 (2), 260–272. doi: 10.1111/J.1471-4159.2010.07100.X 21062286 PMC3042163

[B82] Kaneko N. Kurata M. Yamamoto T. Morikawa S. Masumoto J. (2019). The Role of Interleukin-1 in General Pathology. Inflamm. Regener. 39 (1), 1–16. doi: 10.1186/S41232-019-0101-5 PMC6551897 31182982

[B83] Kapás L. Bohnet S. G. Traynor T. R. Majde J. A. Szentirmai É. Magrath P.. (2008). Spontaneous and Influenza Virus-Induced Sleep are Altered in TNF-α Double-Receptor Deficient Mice. J. Appl. Physiol. 105 (4), 1187–1198. doi: 10.1152/japplphysiol.90388.2008 18687977 PMC2576045

[B84] Kapás L. Fang J. Krueger J. M. (1994). Inhibition of Nitric Oxide Synthesis Inhibits Rat Sleep. Brain Res. 664 (1-2), 189–196. doi: 10.1016/0006-8993(94)91969-0 7534601

[B85] Kapas L. Krueger J. M. (1992). Tumor Necrosis Factor-Beta Induces Sleep, Fever, and Anorexia. Am. J. Physiol. 263 (3 Pt 2), R703–R707. doi: 10.1152/AJPREGU.1992.263.3.R703 1415661

[B86] Kaur C. Ling E. A. (2017). The Circumventricular Organs. Histol. Histopathol. 32 (9), 879–892. doi: 10.14670/HH-11-881 28177105

[B87] Keller M. Mazuch J. Abraham U. Eom G. D. Herzog E. D. Volk H. D.. (2009). A Circadian Clock in Macrophages Controls Inflammatory Immune Responses. Proc. Natl. Acad. Sci. U. S. A. 106 (50), 21407–21412. doi: 10.1073/PNAS.0906361106 19955445 PMC2795539

[B88] Khakh B. S. North R. A. (2012). Neuromodulation by Extracellular ATP and P2X Receptors in the CNS. Neuron 76 (1), 51–69. doi: 10.1016/J.NEURON.2012.09.024 23040806 PMC4064466

[B89] Kim Y. Laposky A. D. Bergmann B. M. Turek F. W. (2007). Repeated Sleep Restriction in Rats Leads to Homeostatic and Allostatic Responses During Recovery Sleep. Proc. Natl. Acad. Sci. U. S. A. 104 (25), 10697–10702. doi: 10.1073/PNAS.0610351104 17548824 PMC1885821

[B90] Kim M. J. Lee J. H. Duffy J. F. (2013). Circadian Rhythm Sleep Disorders. J. Clin. Outcomes. Manage. 20 (11), 513–528. PMC4212693 25368503

[B91] Krueger J. M. Frank M. G. Wisor J. P. Roy S. (2016). Sleep Function: Toward Elucidating an Enigma. Sleep. Med. Rev. 28, 46–54. doi: 10.1016/J.SMRV.2015.08.005 26447948 PMC4769986

[B92] Krueger J. M. Rector D. M. Churchill L. (2007). Sleep and Cytokines. Sleep. Med. Clin. 2 (2), 161–169. doi: 10.1016/J.JSMC.2007.03.003 19098992 PMC2605347

[B93] Krueger J. M. Taishi P. De A. Davis C. J. Winters B. D Clinton J.. (2010). ATP and the Purine Type 2 X7 Receptor Affect Sleep. J. Appl. Physiol. 109 (5), 1318–1327. doi: 10.1152/japplphysiol.00586.2010 20829501 PMC2980381

[B94] Krumm B. Xiang Y. Deng J. (2014). Structural Biology of the IL-1 Superfamily: Key Cytokines in the Regulation of Immune and Inflammatory Responses. Protein Sci. 23 (5), 526–538. doi: 10.1002/PRO.2441 24677376 PMC4005705

[B95] Kubota T. Fang J. Brown R. A. Krueger J. M. (2001). Interleukin-18 Promotes Sleep in Rabbits and Rats. Am. J. Physiol. Regul. Integr. Comp. Physiol. 281 (3): R828–R838. doi: 10.1152/AJPREGU.2001.281.3.R828 11506998

[B96] Kubota T. Fang J. Guan Z. Brown R. A. Krueger J. M. (2001). Vagotomy Attenuates Tumor Necrosis Factor-α-Induced Sleep and EEG δ-Activity in Rats. Am. J. Physiol. - Regul. Integr. Comp. Physiol. 280 (4 49-4), R1213–R1220. doi: 10.1152/ajpregu.2001.280.4.r1213 11247847

[B97] Kubota T. Kushikata T. Fang J. Krueger J. M. (2000). Nuclear factor-kappaB Inhibitor Peptide Inhibits Spontaneous and Interleukin-1beta-Induced Sleep. Am. J. Physiol. Regul. Integr. Comp. Physiol. 279 (2). doi: 10.1152/AJPREGU.2000.279.2.R404 10938226

[B98] Kubota T. Li N. Guan Z. Brown R. A. Krueger J. M. (2002). Intrapreoptic Microinjection of TNF-Alpha Enhances Non-REM Sleep in Rats. Brain Res. 932 (1-2), 37–44. doi: 10.1016/S0006-8993(02)02262-X 11911859

[B99] Kumar S. Rai S. Hsieh K. C. McGinty D. Alam M. N. Szymusiak R. (2013). Adenosine A(2A) Receptors Regulate the Activity of Sleep Regulatory GABAergic Neurons in the Preoptic Hypothalamus. Am. J. Physiol. Regul. Integr. Comp. Physiol. 305 (1), R31–41. doi: 10.1152/AJPREGU.00402.2012 PMC3727028 23637137

[B100] Lai A. Y. Swayze R. D. El-Husseini A. Song C. (2006). Interleukin-1 Beta Modulates AMPA Receptor Expression and Phosphorylation in Hippocampal Neurons. J. Neuroimmunol. 175 (1-2), 97–106. doi: 10.1016/J.JNEUROIM.2006.03.001 16626814

[B101] Larkin J. E. Yokogawa T. Heller H. C. Franken P. Ruby N. F. (2004). Homeostatic Regulation of Sleep in Arrhythmic Siberian Hamsters. Am. J. Physiol. Regul. Integr. Comp. Physiol. 287 (1), , R104-R111. doi: 10.1152/AJPREGU.00676.2003 14962826

[B102] Laye S. Bluthe R. M. Kent S. Combe C. Medina C. Parnet P.. (1995). Subdiaphragmatic Vagotomy Blocks Induction of IL-1β mRNA in Mice Brain in Response to Peripheral LPS. Am. J. Physiol. - Regul. Integr. Comp. Physiol. 268 (5 37-5), R1327–R1331. doi: 10.1152/ajpregu.1995.268.5.r1327 7771597

[B103] Levi F. Schibler U. (2007). Circadian Rhythms: Mechanisms and Therapeutic Implications. Annu. Rev. Pharmacol. Toxicol. 47, 593–628. doi: 10.1146/ANNUREV.PHARMTOX.47.120505.105208 17209800

[B104] Liu T. Zhang L. Joo D. Sun S. C. (2017). NF-κb Signaling in Inflammation. Signal Transduct. Target. Ther. 2, 17023. doi: 10.1038/sigtrans.2017.23 29158945 PMC5661633

[B105] Li D. Wu M. (2021). Pattern Recognition Receptors in Health and Diseases. Signal Transduct. Target. Ther. 6 (1). doi: 10.1038/S41392-021-00687-0 PMC8333067 34344870

[B106] Macvicar B. A. Newman E. A. (2015). Astrocyte Regulation of Blood Flow in the Brain. Cold Spring Harb. Perspect. Biol. 7 (5), 1–15. doi: 10.1101/cshperspect.a020388 PMC4448617 25818565

[B107] Maher C. O. Anderson R. E. Martin H. S. McClelland R. L. Meyer F. B. (2003). Interleukin-1β and Adverse Effects on Cerebral Blood Flow During Long-Term Global Hypoperfusion. J. Neurosurg. 99 (5), 907–912. doi: 10.3171/jns.2003.99.5.0907 14609172

[B108] Makarenkova H. P. Shah S. B. Shestopalov V. I. (2018). The Two Faces of Pannexins: New Roles in Inflammation and Repair. J. Inflamm. Res. 11, 273–288. doi: 10.2147/JIR.S128401 29950881 PMC6016592

[B109] Manfridi A. Brambilla D. Bianchi S. Mariotti M. Opp M. R. Imeri L. (2003). Interleukin-1β Enhances non-Rapid Eye Movement Sleep When Microinjected Into the Dorsal Raphe Nucleus and Inhibits Serotonergic Neurons *In Vitro* . Eur. J. Neurosci. 18 (5), 1041–1049. doi: 10.1046/j.1460-9568.2003.02836.x 12956704

[B110] Masih J. Belschak F. Willem Verbeke J. M. I. (2019). Mood Configurations and Their Relationship to Immune System Responses: Exploring the Relationship Between Moods, Immune System Responses, Thyroid Hormones, and Social Support. PloS One 14 (5). doi: 10.1371/JOURNAL.PONE.0216232 PMC6544341 31150403

[B111] Melikian N. Seddon M. D. Casadei B. Chowienczyk P. J. Shah A. M. (2009). Neuronal Nitric Oxide Synthase and Human Vascular Regulation. Trends Cardiovasc. Med. 19 (8), 256–262. doi: 10.1016/J.TCM.2010.02.007 20447567 PMC2984617

[B112] Mistlberger R. E. Bergmann B. M. Waldenar W. Rechtschaffen A. (1983). Recovery Sleep Following Sleep Deprivation in Intact and Suprachiasmatic Nuclei-Lesioned Rats. Sleep 6 (3), 217–233. doi: 10.1093/SLEEP/6.3.217 6622879

[B113] Mistlberger RE M. E. (1995). Computational and Entrainment Models of Circadian Food-Anticipatory Activity: Evidence From non-24-Hr Feeding Schedules - PubMed. Behav. Neurosci. 109 (4), 790–798. doi: 7576223 doi: 10.1037/0735-7044.109.4.790 7576223

[B114] Mitsui S. Yamaguchi S. Matsuo T. Ishida Y. Okamura H. (2001). Antagonistic Role of E4BP4 and PAR Proteins in the Circadian Oscillatory Mechanism. Genes Dev. 15 (8), 995–1006. doi: 10.1101/GAD.873501 11316793 PMC312673

[B115] Miyata S. (2015). New Aspects in Fenestrated Capillary and Tissue Dynamics in the Sensory Circumventricular Organs of Adult Brains. Front. Neurosci. 9, 390. doi: 10.3389/fnins.2015.00390 26578857 PMC4621430

[B116] Moon J. S. Hisata S. Park M. A. DeNicola G. M. Ryter S. W. Nakahira K.. (2015). MTORC1-Induced HK1-Dependent Glycolysis Regulates NLRP3 Inflammasome Activation. Cell Rep. 12 (1), 102–115. doi: 10.1016/j.celrep.2015.05.046 26119735 PMC4858438

[B117] Moore R. Y. Halaris A. E. Jones B. E. (1978). Serotonin Neurons of the Midbrain Raphe: Ascending Projections. J. Comp. Neurol. 180 (3), 417–438. doi: 10.1002/CNE.901800302 77865

[B118] Morairty S. R. Dittrich L. Pasumarthi R. K. Valladao D. Heiss J. E. Gerashchenko D.. (2013). A Role for Cortical nNOS/NK1 Neurons in Coupling Homeostatic Sleep Drive to EEG Slow Wave Activity. Proc. Natl. Acad. Sci. U. S. A. 110 (50), 20272–20277. doi: 10.1073/pnas.1314762110 24191004 PMC3864296

[B119] Muindi F. Zeitzer J. M. Heller H. C. (2014). Retino-Hypothalamic Regulation of Light-Induced Murine Sleep. Front. Syst. Neurosci. 8. doi: 10.3389/FNSYS.2014.00135 PMC4121530 25140132

[B120] Murphy P. J. Badia P. Myers B. L. Boecker M. R. Wright K. P. (1994). Nonsteroidal Anti-Inflammatory Drugs Affect Normal Sleep Patterns in Humans. Physiol. Behav. 55 (6), 1063–1066. doi: 10.1016/0031-9384(94)90388-3 8047572

[B121] Naylor E. Bergmann B. M. Krauski K. Zee P. C. Takahashi J. S. Vitaterna M. H.. (2000). The Circadian Clock Mutation Alters Sleep Homeostasis in the Mouse. J. Neurosci. 20 (21), 8138–8143. doi: 10.1523/JNEUROSCI.20-21-08138.2000 11050136 PMC6772726

[B122] Nguyen J. Gibbons C. M. Dykstra-Aiello C. Ellingsen R. Koh K. M. S. Taishi P.. (2019). Interleukin-1 Receptor Accessory Proteins Are Required for Normal Homeostatic Responses to Sleep Deprivation. J. Appl. Physiol. 127 (3), 770–780. doi: 10.1152/japplphysiol.00366.2019 31295066 PMC6766708

[B123] Nicholson C. Hrabětová S. (2017). Brain Extracellular Space: The Final Frontier of Neuroscience. Biophys. J. 113 (10), 2133–2142. doi: 10.1016/J.BPJ.2017.06.052 28755756 PMC5700249

[B124] Obal F. Opp M. Cady A. B. Johannsen L. Postlethwaite A. E. Poppleton H. M.. (1990). Interleukin 1 Alpha and an Interleukin 1 Beta Fragment are Somnogenic. Am. J. Physiol. 259 (3 Pt 2), R439–R446. doi: 10.1152/AJPREGU.1990.259.3.R439 2396703

[B125] Oeckinghaus A. Ghosh S. (2009). The NF-kappaB Family of Transcription Factors and its Regulation. Cold Spring Harb. Perspect. Biol. 1 (4). doi: 10.1101/CSHPERSPECT.A000034 PMC2773619 20066092

[B126] Oles V. Koh K. M. S. Dykstra-Aiello C. J. Savenkova M. Gibbons C. M. Nguyen J. T.. (2020). Sleep- and Time of Day-Linked RNA Transcript Expression in Wild-Type and IL1 Receptor Accessory Protein-Null Mice. J. Appl. Physiol. 128 (6), 1506–1522. doi: 10.1152/JAPPLPHYSIOL.00839.2019 32324480 PMC7311685

[B127] Onoe H. Watanabe Y. Ono K. Koyama Y. Hayaishi O. (1992). Prostaglandin E2 Exerts an Awaking Effect in the Posterior Hypothalamus at a Site Distinct From That Mediating its Febrile Action in the Anterior Hypothalamus. J. Neurosci. 12 (7), 2715–2725. doi: 10.1523/JNEUROSCI.12-07-02715.1992 1613554 PMC6575852

[B128] Oppenheim J. J. (2001). Cytokines: Past, Present, and Future. Int. J. Hematol. 74 (1), 3–8. doi: 10.1007/BF02982543 11530802

[B129] Opp M. R. Krueger J. M. (1991). Interleukin 1-Receptor Antagonist Blocks Interleukin 1-Induced Sleep and Fever. Am. J. Physiol. 260 (2 Pt 2), R453–R457. doi: 10.1152/AJPREGU.1991.260.2.R453 1825458

[B130] Opp M. R. Krueger J. M. (2015). Sleep and Immunity: A Growing Field With Clinical Impact. Brain Behav. Immun. 47, 1–3. doi: 10.1016/J.BBI.2015.03.011 25849976 PMC4685944

[B131] Patke A. Young M. W. Axelrod S. (2020). Molecular Mechanisms and Physiological Importance of Circadian Rhythms. Nat. Rev. Mol. Cell Biol. 21 (2), 67–84. doi: 10.1038/S41580-019-0179-2 31768006

[B132] Pavlov V. A. Tracey K. J. (2012). The Vagus Nerve and the Inflammatory Reflex - Linking Immunity and Metabolism. Nat. Rev. Endocrinol. 8 (12), 743–754. doi: 10.1038/nrendo.2012.189 23169440 PMC4082307

[B133] Pelegrin P. (2021). P2X7 Receptor and the NLRP3 Inflammasome: Partners in Crime. Biochem. Pharmacol. 187, 114385. doi: 10.1016/J.BCP.2020.114385 33359010

[B134] Pellegrini C. Antonioli L. Lopez-Castejon G. Blandizzi C. Fornai M. (2017). Canonical and Non-Canonical Activation of NLRP3 Inflammasome at the Crossroad Between Immune Tolerance and Intestinal Inflammation. Front. Immunol. 8. doi: 10.3389/FIMMU.2017.00036 PMC5263152 28179906

[B135] Pickel L. Sung H. K. (2020). Feeding Rhythms and the Circadian Regulation of Metabolism. Front. Nutr 7. doi: 10.3389/FNUT.2020.00039 PMC7182033 32363197

[B136] Pols T. Singh S. Deelman-Driessen C. Gaastra B. F. Poolman B. (2021). Enzymology of the Pathway for ATP Production by Arginine Breakdown. FEBS J. 288 (1), 293–309. doi: 10.1111/FEBS.15337 32306469 PMC7818446

[B137] Porkka-Heiskanen T. Strecker R. E. McCarley R. W. (2000). Brain Site-Specificity of Extracellular Adenosine Concentration Changes During Sleep Deprivation and Spontaneous Sleep: An *In Vivo* Microdialysis Study. Neuroscience 99 (3), 507–517. doi: 10.1016/S0306-4522(00)00220-7 11029542

[B138] Porkka-Heiskanen T. Strecker R. E. Thakkar M. Bjørkum A. A. Greene R. W. McCarley R. W. (1997). Adenosine: A Mediator of the Sleep-Inducing Effects of Prolonged Wakefulness. Science 276 (5316), 1265–1267. doi: 10.1126/SCIENCE.276.5316.1265 9157887 PMC3599777

[B139] Preitner N. Damiola F. Lopez-Molina L. Zakany J. Duboule D. Albrecht U.. (2002). The Orphan Nuclear Receptor REV-ERBalpha Controls Circadian Transcription Within the Positive Limb of the Mammalian Circadian Oscillator. Cell 110 (2), 251–260. doi: 10.1016/S0092-8674(02)00825-5 12150932

[B140] Probert L. (2015). TNF and its Receptors in the CNS: The Essential, the Desirable and the Deleterious Effects. Neuroscience 302, 2–22. doi: 10.1016/J.NEUROSCIENCE.2015.06.038 26117714

[B141] Ramesh V. Thatte H. S. McCarley R. W. Basheer R. (2007). Adenosine and Sleep Deprivation Promote NF-kappaB Nuclear Translocation in Cholinergic Basal Forebrain. J. Neurochem. 100 (5), 1351–1363. doi: 10.1111/J.1471-4159.2006.04314.X 17316404

[B142] Ramkumar V. Jhaveri K. A. Xie X. Jajoo S. Toth L. A. (2011). Nuclear Factor κb and Adenosine Receptors: Biochemical and Behavioral Profiling. Curr. Neuropharmacol. 9 (2), 342–349. doi: 10.2174/157015911795596559 22131942 PMC3131724

[B143] Refinetti R. (2010). Entrainment of Circadian Rhythm by Ambient Temperature Cycles in Mice. J. Biol. Rhythms. 25 (4), 247–256. doi: 10.1177/0748730410372074 20679494

[B144] Reilly D. F. Curtis A. M. Cheng Y. Westgate E. J. Rudic R. D. Paschos G.. (2008). Peripheral Circadian Clock Rhythmicity is Retained in the Absence of Adrenergic Signaling. Arterioscler. Thromb. Vasc. Biol. 28 (1), 121–126. doi: 10.1161/ATVBAHA.107.152538 17975121 PMC2752700

[B145] Reutrakul S. Knutson K. L. (2015). Consequences of Circadian Disruption on Cardiometabolic Health. Sleep. Med. Clin. 10 (4), 455–468. doi: 10.1016/J.JSMC.2015.07.005 26568122 PMC4648711

[B146] Ricciotti E. FitzGerald G. A. (2011). Prostaglandins and Inflammation. Arterioscler. Thromb. Vasc. Biol. 31 (5), 986–1000. doi: 10.1161/ATVBAHA.110.207449 21508345 PMC3081099

[B147] Riera Romo M. Pérez-Martínez D. Castillo Ferrer C. (2016). Innate Immunity in Vertebrates: An Overview. Immunology 148 (2), 125–139. doi: 10.1111/IMM.12597 26878338 PMC4863567

[B148] Rindflesch T. C. Blake C. L. Cairelli M. J. Fiszman M. Zeiss C. J. Kilicoglu H. (2018). Investigating the Role of Interleukin-1 Beta and Glutamate in Inflammatory Bowel Disease and Epilepsy Using Discovery Browsing. J. BioMed. Semantics. 9 (1). doi: 10.1186/S13326-018-0192-Y PMC6307110 30587224

[B149] Rockstrom M. D. Chen L. Taishi P. Nguyen J. T. Gibbons C. M. Veasey S. C.. (2018). Tumor Necrosis Factor Alpha in Sleep Regulation. Sleep. Med. Rev. 40, 69–78. doi: 10.1016/j.smrv.2017.10.005 29153862 PMC5955790

[B150] Saint-Mleux B. Bayer L. Eggermann E. Jones B. E. Mühlethaler M. Serafin M. (2007). Suprachiasmatic Modulation of Noradrenaline Release in the Ventrolateral Preoptic Nucleus. J. Neurosci. 27 (24), 6412–6416. doi: 10.1523/JNEUROSCI.1432-07.2007 17567801 PMC6672428

[B151] Sato T. K. Panda S. Miraglia L. J. Reyes T. M. Rudic R. D. McNamara P.. (2004). A Functional Genomics Strategy Reveals Rora as a Component of the Mammalian Circadian Clock. Neuron 43 (4), 527–537. doi: 10.1016/J.NEURON.2004.07.018 15312651

[B152] Scammell T. E. Gerashchenko D. Y. Mochizuki T. McCarthy M. T. Estabrooke I. V. Sears C. A.. (2001). An Adenosine A2a Agonist Increases Sleep and Induces Fos in Ventrolateral Preoptic Neurons. Neuroscience 107 (4), 653–663. doi: 10.1016/S0306-4522(01)00383-9 11720788

[B153] Sedger L. M. McDermott M. F. (2014). TNF and TNF-Receptors: From Mediators of Cell Death and Inflammation to Therapeutic Giants - Past, Present and Future. Cytokine Growth Factor. Rev. 25 (4), 453–472. doi: 10.1016/J.CYTOGFR.2014.07.016 25169849

[B154] Shiromani P. J. Xu M. Winston E. M. Shiromani S. N. Gerashchenko D. Weaver D. R. (2004). Sleep Rhythmicity and Homeostasis in Mice With Targeted Disruption of Mperiod Genes. Am. J. Physiol. - Regul. Integr. Comp. Physiol. 287 (1 56-1), 47–57. doi: 10.1152/AJPREGU.00138.2004/ASSET/IMAGES/LARGE/ZH60070422500008.JPEG 15031135

[B155] Shoham S. Davenne D. Cady A. B. Dinarello C. A. Krueger J. M. (1987). Recombinant Tumor Necrosis Factor and Interleukin 1 Enhance Slow-Wave Sleep. Am. J. Physiol. - Regul. Integr. Comp. Physiol. 253(1 (1 (22/1), R142–R149. doi: 10.1152/ajpregu.1987.253.1.r142 3496800

[B156] Simon L. S. (1999). Role and Regulation of Cyclooxygenase-2 During Inflammation. Am. J. Med. 106 (5B), 37S–42S. doi: 10.1016/S0002-9343(99)00115-1 10390126

[B157] Sollberger G. Strittmatter G. E. Garstkiewicz M. Sand J. Beer H. D. (2014). Caspase-1: The Inflammasome and Beyond. Innate. Immun. 20 (2), 115–125. doi: 10.1177/1753425913484374 23676582

[B158] Stellwagen D. Malenka R. C. (2006). Synaptic Scaling Mediated by Glial TNF-Alpha. Nature 440 (7087), 1054–1059. doi: 10.1038/NATURE04671 16547515

[B159] Swamy M. Salleh M. J. M. Sirajudeen K. N. S. Yusof W. R. W. Chandran G. (2010). Nitric Oxide (No), Citrulline - No Cycle Enzymes, Glutamine Synthetase and Oxidative Stress in Anoxia (Hypobaric Hypoxia) and Reperfusion in Rat Brain. Int. J. Med. Sci. 7 (3), 147–154. doi: 10.7150/IJMS.7.147 20567615 PMC2880843

[B160] Syková E. Nicholson C. (2008). Diffusion in Brain Extracellular Space. Physiol. Rev. 88 (4), 1277–1340. doi: 10.1152/PHYSREV.00027.2007 18923183 PMC2785730

[B161] Szentirmai É. Kapás L. (2019). Sleep and Body Temperature in Tnfα Knockout Mice: The Effects of Sleep Deprivation, β3-AR Stimulation and Exogenous Tnfα. Brain Behav. Immun. 81, 260–271. doi: 10.1016/j.bbi.2019.06.022 31220563 PMC6754767

[B162] Taishi P. Davis C. J. Bayomy O. Zielinski M. R. Liao F. Clinton J. M.. (2012). Brain-Specific Interleukin-1 Receptor Accessory Protein in Sleep Regulation. J. Appl. Physiol. 112 (6), 1015–1022. doi: 10.1152/japplphysiol.01307.2011 22174404 PMC3311656

[B163] Takahashi J. S. (2017). Transcriptional Architecture of the Mammalian Circadian Clock. Nat. Rev. Genet. 18 (3), 164–179. doi: 10.1038/NRG.2016.150 27990019 PMC5501165

[B164] Takahashi S. Kapa s L Fang J. Krueger J. M. (1995). An Anti-Tumor Necrosis Factor Antibody Suppresses Sleep in Rats and Rabbits. Brain Res. 690 (2), 241–244. doi: 10.1016/0006-8993(95)00609-T 8535843

[B165] Takahashi S. Kapás L. Fang J. Krueger J. M. (1999). Somnogenic Relationships Between Tumor Necrosis Factor and Interleukin- 1. Am. J. Physiol. - Regul. Integr. Comp. Physiol. 276 (4 45-4), R1132–R1140. doi: 10.1152/ajpregu.1999.276.4.r1132 10198395

[B166] Takahashi S. Kapás L. Krueger J. M. (1996). A Tumor Necrosis Factor (TNF) Receptor Fragment Attenuates TNF-Alpha- and Muramyl Dipeptide-Induced Sleep and Fever in Rabbits. J. Sleep. Res. 5 (2), 106–114. doi: 10.1046/J.1365-2869.1996.D01-63.X 8795811

[B167] Temel S. G. Kahveci Z. (2009). Cyclooxygenase-2 Expression in Astrocytes and Microglia in Human Oligodendroglioma and Astrocytoma. J. Mol. Histol. 40 (5-6), 369–377. doi: 10.1007/S10735-009-9250-1 20052522

[B168] Timmons G. A. Carroll R. G. O’Siorain J. R. Cervantes-Silva M. P. Fagan L. E. Cox S. L.. (2021). The Circadian Clock Protein BMAL1 Acts as a Metabolic Sensor In Macrophages to Control the Production of Pro IL-1β. Front. Immunol. 12. doi: 10.3389/FIMMU.2021.700431 PMC8630747 34858390

[B169] Tobler I. Borbély A. A. Groos G. (1983). The Effect of Sleep Deprivation on Sleep in Rats With Suprachiasmatic Lesions. Neurosci. Lett. 42 (1), 49–54. doi: 10.1016/0304-3940(83)90420-2 6657146

[B170] Trachsel L. Edgar D. M. Seidel W. F. Craig Heller H. (1992). Sleep Homeostasis in Suprachiasmatic Nuclei-Lesioned Rats: Effects of Sleep Deprivation and Triazolam Administration. Brain Res. 589 (2), 253–261. doi: 10.1016/0006-8993(92)91284-L 1393593

[B171] Tracy R. P. (2006). The Five Cardinal Signs of Inflammation: Calor, Dolor, Rubor, Tumor … and Penuria (Apologies to Aulus Cornelius Celsus, De Medicina, C. A.D. 25). J. Gerontol. A. Biol. Sci. Med. Sci. 61 (10), 1051–1052. doi: 10.1093/GERONA/61.10.1051 17077197

[B172] Tsai P. S. Kaufhold J. P. Blinder P. Friedman B. Drew P. J. Karten H. J.. (2009). Correlations of Neuronal and Microvascular Densities in Murine Cortex Revealed by Direct Counting and Colocalization of Nuclei and Vessels. J. Neurosci. 29 (46), 14553–14570. doi: 10.1523/JNEUROSCI.3287-09.2009 19923289 PMC4972024

[B173] Turner K. L. Gheres K. W. Proctor E. A. Drew P. J. (2020). Neurovascular Coupling and Bilateral Connectivity During Nrem and Rem Sleep. Elife 9, 1. doi: 10.7554/eLife.62071 PMC7758068 33118932

[B174] Tüshaus L. Omlin X. Tuura R. O. G. Federspiel A. Luechinger R. Staempfli P.. (2017). In Human Non-REM Sleep, More Slow-Wave Activity Leads to Less Blood Flow in the Prefrontal Cortex. Sci. Rep. 7 (1), 14993. doi: 10.1038/s41598-017-12890-7 29101338 PMC5670199

[B175] Urade Y. Eguchi N. Qu W. M. Sakata M. Huang Z. L. Chen J. -F.. (2003). Sleep Regulation in Adenosine A2A Receptor-Deficient Mice. Neurology 61 (11 Suppl 6), S94–6. doi: 10.1212/01.WNL.0000095222.41066.5E 14663019

[B176] van Diepen H. C. Lucassen E. A. Yasenkov R. Groenen I. Ijzerman A. P. Meijer J. H.. (2014). Caffeine Increases Light Responsiveness of the Mouse Circadian Pacemaker. Eur. J. Neurosci. 40 (10), 3504–3511. doi: 10.1111/EJN.12715 25196050

[B177] Viola-Saltzman M. Watson N. F. (2012). Traumatic Brain Injury and Sleep Disorders. Neurol. Clin. 30 (4), 1299–1312. doi: 10.1016/J.NCL.2012.08.008 23099139 PMC3482689

[B178] Visan I. (2019). Mapping IL-1 in the Brain. Nat. Immunol. 20 (3), 245. doi: 10.1038/S41590-019-0337-X 30778247

[B179] Voet S. Srinivasan S. Lamkanfi M. Loo G. (2019). Inflammasomes in Neuroinflammatory and Neurodegenerative Diseases. EMBO Mol. Med. 11 (6), e10248. doi: 10.15252/emmm.201810248 31015277 PMC6554670

[B180] Wajant H. Siegmund D. (2019). TNFR1 and TNFR2 in the Control of the Life and Death Balance of Macrophages. Front. Cell Dev. Biol. 7. doi: 10.3389/fcell.2019.00091 PMC6548990 31192209

[B181] Waldmann T. A. (2018). Cytokines in Cancer Immunotherapy. Cold Spring Harb. Perspect. Biol. 10 (12). doi: 10.1101/CSHPERSPECT.A028472 PMC6280701 29101107

[B182] Wang G. Gilbert J. Man H. Y. (2012). AMPA Receptor Trafficking in Homeostatic Synaptic Plasticity: Functional Molecules and Signaling Cascades. Neural Plast. 2012. doi: 10.1155/2012/825364 PMC3359728 22655210

[B183] Williams J. W. Huang L. H. Randolph G. J. (2019). Cytokine Circuits in Cardiovascular Disease. Immunity 50 (4), 941–954. doi: 10.1016/J.IMMUNI.2019.03.007 30995508 PMC6924925

[B184] Williams S. D. Lewis L. D. (2020). Blood Flow Supplying the Sleeping Brain. Elife 9, 1–3. doi: 10.7554/ELIFE.64597 PMC7758057 33355090

[B185] Wisor J. P. O’Hara B. F. Terao A. Selby C. P. Kilduff T. S. Sancar A.. (2002). A Role for Cryptochromes in Sleep Regulation. BMC Neurosci. 3. doi: 10.1186/1471-2202-3-20 PMC149230 12495442

[B186] Xie L. Kang H. Xu Q. Chen M. J. Liao Y. Thiyagarajan M.. (2013). Sleep Drives Metabolite Clearance From the Adult Brain. Science (80-) 342 (6156), 373–377. doi: 10.1126/science.1241224 PMC3880190 24136970

[B187] Xu D. Mu R. Wei X. (2019). The Roles of IL-1 Family Cytokines in the Pathogenesis of Systemic Sclerosis. Front. Immunol. 10. doi: 10.3389/FIMMU.2019.02025 PMC6753625 31572353

[B188] Ye L. Huang Y. Zhao L. Li Y. Sun L. Zhou Y.. (2013). IL-1β and TNF-α Induce Neurotoxicity Through Glutamate Production: A Potential Role for Neuronal Glutaminase. J. Neurochem. 125 (6), 897–908. doi: 10.1111/JNC.12263 23578284 PMC3747774

[B189] Yoshida H. Kubota T. Krueger J. M. (2003). A Cyclooxygenase-2 Inhibitor Attenuates Spontaneous and TNF-Alpha-Induced non-Rapid Eye Movement Sleep in Rabbits. Am. J. Physiol. Regul. Integr. Comp. Physiol. 285 (1), R99–R109. doi: 10.1152/AJPREGU.00609.2002 12623776

[B190] Yoshida Y. Matsumura H. Nakajima T. Mandai M. Urakami T. Kuroda K.. (2000). Prostaglandin E (EP) Receptor Subtypes and Sleep: Promotion by EP4 and Inhibition by EP1/EP2. Neuroreport 11 (10), 2127–2131. doi: 10.1097/00001756-200007140-00014 10923657

[B191] Young M. W. Kay S. A. (2001). Time Zones: A Comparative Genetics of Circadian Clocks. Nat. Rev. Genet. 2 (9), 702–715. doi: 10.1038/35088576 11533719

[B192] Zamir M. Moir M. E. Klassen S. A. Balestrini C. S. Shoemaker J. K. (2018). Cerebrovascular Compliance Within the Rigid Confines of the Skull. Front. Physiol. 9. doi: 10.3389/FPHYS.2018.00940 PMC6056744 30065667

[B193] Zhou R. Tardivel A. Thorens B. Choi I. Tschopp J. (2010). Thioredoxin-Interacting Protein Links Oxidative Stress to Inflammasome Activation. Nat. Immunol. 11 (2), 136–140. doi: 10.1038/ni.1831 20023662

[B194] Zielinski M. R. Atochin D. N. McNally J. M. McKenna J. T. Huang P. L. Strecker R. E.. (2019). Somatostatin+/nNOS+ Neurons are Involved in Delta Electroencephalogram Activity and Corticaldependent Recognition Memory. Sleep 42 (10), 1827. doi: 10.1093/sleep/zsz143 PMC6783898 31328777

[B195] Zielinski M. R. Dunbrasky D. L. Taishi P. Souza G. Krueger J. M. (2013). Vagotomy Attenuates Brain Cytokines and Sleep Induced by Peripherally Administered Tumor Necrosis Factor-α and Lipopolysaccharide in Mice. Sleep 36 (8), 1227–1238. doi: 10.5665/sleep.2892 23904683 PMC3700720

[B196] Zielinski M. R. Gerashchenko D. Karpova S. A. Konanki V. McCarley R. W. Sutterwala F. S.. (2017). The NLRP3 Inflammasome Modulates Sleep and NREM Sleep Delta Power Induced by Spontaneous Wakefulness, Sleep Deprivation and Lipopolysaccharide. Brain Behav. Immun. 62, 137–150. doi: 10.1016/j.bbi.2017.01.012 28109896 PMC5373953

[B197] Zielinski M. R. Gerashchenko D. Patel D. Torres K. Desrosiers G. (2019). 0219 Mice Lacking IL-18 Have Reduced Sleep and Slow-Waveactivityresponses to Sleep Promoting Stimuli. Sleep 42 (Supplement_1), A90–A90. doi: 10.1093/SLEEP/ZSZ067.218

[B198] Zielinski M. R. Karpova S. A. Yang X. Gerashchenko D. (2015). Substance P and the Neurokinin-1 Receptor Regulate Electroencephalogram non-Rapid Eye Movement Sleep Slow-Wave Activity Locally. Neuroscience 284, 260–272. doi: 10.1016/j.neuroscience.2014.08.062 25301750 PMC4268367

[B199] Zielinski M. R. Kim Y. Karpova S. A. Winston S. McCarley R. W. Strecker R. E.. (2013). Sleep Active Cortical Neurons Expressing Neuronal Nitric Oxide Synthase are Active After Both Acute Sleep Deprivation and Chronic Sleep Restriction. Neuroscience 247, 35–42. doi: 10.1016/j.neuroscience.2013.05.013 23685166 PMC3801181

[B200] Zielinski M. R. Kim Y. Karpova S. A. McCarley R. W. Strecker R. E. Gerashchenko D. (2014). Chronic Sleep Restriction Elevates Brain Interleukin-1 Beta and Tumor Necrosis Factor-Alpha and Attenuates Brain-Derived Neurotrophic Factor Expression. Neurosci. Lett. 580, 27–31. doi: 10.1016/j.neulet.2014.07.043 25093703 PMC4162816

[B201] Zielinski M. R. Krueger J. M. (2011). Sleep and Innate Immunity. Front. Biosci. - Sch. 3 S (2), 632–642. doi: 10.2741/s176 PMC3645929 21196401

[B202] Zielinski M. R. McKenna J. T. McCarley R. W. (2016). Functions and Mechanisms of Sleep. AIMS. Neurosci. 3 (1), 67–104. doi: 10.3934/Neuroscience.2016.1.67 28413828 PMC5390528

[B203] Zielinski M. R. Souza G. Taishi P. Bohnet S. G. Krueger J. M. (2013). Olfactory Bulb and Hypothalamic Acute-Phase Responses to Influenza Virus: Effects of Immunization. Neuroimmunomodulation 20 (6), 323–333. doi: 10.1159/000351716 23948712 PMC3874867

[B204] Zielinski M. R. Systrom D. M. Rose N. R. (2019). Fatigue, Sleep, and Autoimmune and Related Disorders. Front. Immunol. 10. doi: 10.3389/fimmu.2019.01827 PMC6691096 31447842

[B205] Zielinski M. R. Taishi P. Clinton J. M. Krueger J. M. (2012). 5′-Ectonucleotidase-Knockout Mice Lack non-REM Sleep Responses to Sleep Deprivation. Eur. J. Neurosci. 35 (11), 1789–1798. doi: 10.1111/j.1460-9568.2012.08112.x 22540145 PMC3370120

[B206] Zumkehr J. Rodriguez-Ortiz C. J. Medeiros R. Kitazawa M. (2018). Inflammatory Cytokine, IL-1β, Regulates Glial Glutamate Transporter *via* microRNA-181a *In Vitro* . J. Alzheimers Dis. 63 (3), 965–975. doi: 10.3233/JAD-170828 29710703 PMC7325598

